# Compositions of iron-meteorite parent bodies constrain the structure of the protoplanetary disk

**DOI:** 10.1073/pnas.2306995121

**Published:** 2024-05-28

**Authors:** Bidong Zhang, Nancy L. Chabot, Alan E. Rubin

**Affiliations:** ^a^Department of Earth, Planetary and Space Sciences, University of California, Los Angeles, CA 90095-1567; ^b^Space Exploration Sector, Johns Hopkins University Applied Physics Laboratory, Laurel, MD 20723; ^c^Maine Mineral and Gem Museum, Bethel, ME 04217

**Keywords:** iron meteorites, asteroids, protoplanetary disk, planetesimals

## Abstract

Magmatic iron-meteorite parent bodies are the earliest planetesimals in the Solar System, and they preserve information about conditions and planet-forming processes in the solar nebula. In this study, we include comprehensive elemental compositions and fractional-crystallization modeling for iron meteorites from the cores of five differentiated asteroids from the inner Solar System. Together with previous results of metallic cores from the outer Solar System, we conclude that asteroidal cores from the outer Solar System have smaller sizes, elevated siderophile-element abundances, and simpler crystallization processes than those from the inner Solar System. These differences are related to the formation locations of the parent asteroids because the solar protoplanetary disk varied in redox conditions, elemental distributions, and dynamics at different heliocentric distances. Using highly siderophile-element data from iron meteorites, we reconstruct the distribution of calcium-aluminum-rich inclusions (CAIs) across the protoplanetary disk within the first million years of Solar-System history. CAIs, the first solids to condense in the Solar System, formed close to the Sun. They were, however, concentrated within the outer disk and depleted within the inner disk. Future models of the structure and evolution of the protoplanetary disk should account for this distribution pattern of CAIs.

## Significance

Magmatic iron meteorites are remnants of the metallic cores of the earliest asteroids in our Solar System. These meteorites can provide important information about chemical conditions and protoplanetary disk evolution in early Solar-System history. This work combines compositional analyses and numerical modeling to reconstruct the behavior of elements, bulk compositions, and crystallization processes of asteroidal cores across the Solar System. We find that the diversity of iron-meteorite compositions, crystallization processes, and morphology is ultimately determined by their formation locations within the protoplanetary disk. Ultimately, the distribution pattern of refractory siderophile elements poses an important constraint on disk evolution models.

Nucleosynthetic isotopic compositions (such as Mo, Ru, Ti, Cr, Ni, and W) of stony and stony-iron meteorites show they may have formed in two distinct compositional reservoirs (1–7). This isotopic dichotomy is due to the enrichment of rapid neutron capture process (*r*-process) nuclides in carbonaceous-chondrite-type (CC) materials (carbonaceous chondrites and Eagle Station pallasites) relative to noncarbonaceous-chondrite-type (NC) materials (ordinary and enstatite chondrites, main-group pallasites, mesosiderites, howardite-eucrite-diogenite meteorites, angrites, ureilites, terrestrial rocks, lunar samples, and martian meteorites). Such an isotopic dichotomy is also observed in iron meteorites (5, 8, 9). The nucleosynthetic anomalies are especially well demonstrated on the ε^95^Mo versus ε^94^Mo diagram, where the vast majority of meteorites plot along two parallel *s*-process (slow neutron capture process) mixing lines (10). The CC suite is suggested to be derived from the outer Solar System and the NC suite from the inner Solar System (4, 5). The isotopic dichotomy may result from the separation of the CC and NC reservoirs induced by the early and rapid formation of proto-Jupiter (5, 11) or by a pressure maximum close to proto-Jupiter in a ring-structured protoplanetary disk (12–16).

Iron meteorites are samples of the earliest metallic melts in the Solar System. They are primarily composed of siderophile elements (those such as Ni and Co that partition into metal) and chalcophile elements (those such as Cu and Cr that partition into sulfide). Most iron meteorites in our collections are believed to be derived from metallic cores of the earliest-differentiated asteroids (5, 17). These are labeled magmatic iron meteorites. There are 11 magmatic iron-meteorite groups and one grouplet identified in current meteorite collections. Groups IC, IIAB+IIG, IIIAB, IIIE, and IVA are from the NC reservoir, while groups IIC, IID, IIF, IIIF, IVB, and the South Byron Trio (SBT) are from the CC reservoir (7–9). Some other iron meteorite groups (IAB and IIE) are thought to have crystallized from metallic pools close to or on the asteroids’ surface (18, 19); these groups are classified as nonmagmatic iron meteorites. Both nonmagmatic iron groups are from the NC reservoir, and they crystallized later than the magmatic irons. This study will focus on magmatic irons.

Each magmatic group/grouplet originated within a unique metallic core, except groups IIAB and IIG which were derived from immiscible layers of a single core (20). The formation of magmatic iron-meteorite parent bodies predates the formation of undifferentiated chondrite parent bodies (21, 22). The parent bodies of all magmatic-iron groups accreted <1 Ma after CAI (calcium-aluminum-rich inclusion) formation (5, 17), an event that defines the beginning of Solar-System history. The NC iron groups differentiated slightly earlier than the CC iron groups plus the SBT, but all these groups differentiated within 1 to 4 Ma after CAI formation (5, 17). Thus, magmatic iron-meteorite parent bodies can provide insight into the composition and evolution of metallic melts in the early Solar System. The remnants of these earliest planetesimals also preserve important conditions and processes in the solar nebula that preceded planet formation.

Chemical trends show these magmatic groups fractionally crystallized from the metallic cores of their respective parent bodies (23–25). The bulk compositions and crystallization processes of these asteroidal cores can be reconstructed using fractional-crystallization modeling (23, 26–28), but previous studies focused on a few elements among groups or on many elements within a single group (29–43). Zhang et al. (44) were the first to examine many elements (up to 19) for all CC-iron groups in a single study. Their preliminary conclusion was that the CC-iron cores had more efficient convection, elevated highly siderophile-element (HSE) abundances, and lower bulk S contents.

Our previous study (44) also showed that the CAI distribution in the outer disk at <1 Ma was heterogeneous. The precursor materials of CC magmatic iron-meteorite parent bodies had varying CAI modal abundances (0 to 26 wt.%) (44), indicating the heterogeneous distribution of CAIs in the nebula predated the agglomeration of chondrites. The preservation of the heterogeneous distribution of CAIs in the protoplanetary disk prior to chondrite agglomeration [the so-called CAI storage problem (45–47)] is an important constraint on models of disk dynamics. CAIs are thought to have formed close to the Sun, but it is unclear how they were distributed throughout the protoplanetary disk and why they were enriched in carbonaceous chondrites accreting at large heliocentric distances (44, 45). A possible explanation is that CAIs were transported to the outer Solar System by disk outflow (8) and that the formation of proto-Jupiter created a pressure bump that blocked the CAIs from spiraling back toward the Sun (11). The pressure bump remained efficient in trapping CAIs in the outer Solar System by the time chondrites formed (11).

In this study, we present compositional data for groups IC and IIIE, as well as for some IIAB and IVA irons, and use published compositional data (29, 38, 42, 43, 48, 49). We also use recently revised experimental fractional-crystallization parameters (50) and an updated fractional-crystallization model (40) to reconstruct the bulk compositions (up to 19 elements) and crystallization processes of all five NC iron-meteorite cores. We compare all known CC- and NC-iron cores across the Solar System and examine whether the formation locations of their parent asteroids affected core compositions and crystallization processes. We also estimate the CAI distribution across the protoplanetary disk within the first million years of Solar-System history to provide additional constraints on disk evolution models.

## Results

### Group IC.

**Fig. 1.**
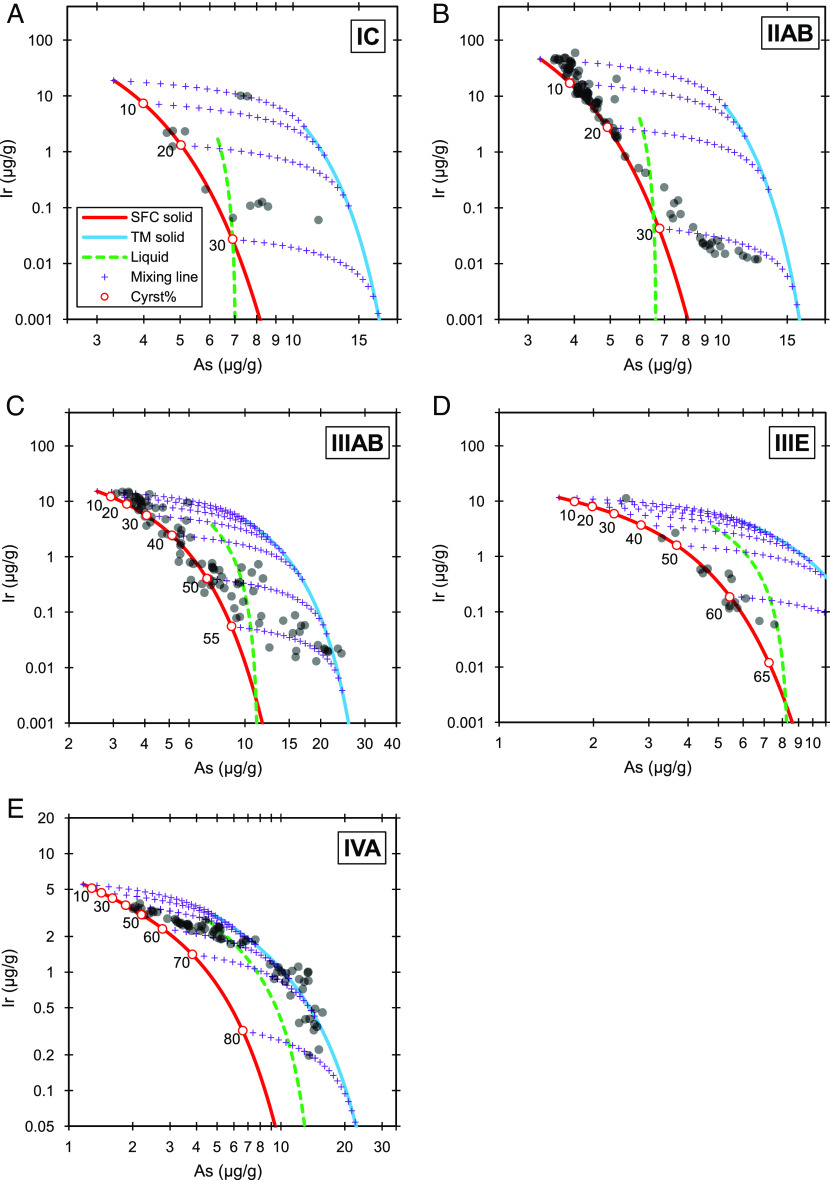
Fractional crystallization modeling of Ir-As trends for iron-meteorite groups: (*A*) IC, (*B*) IIAB, (*C*) IIIAB, (*D*) IIIE, and (*E*) IVA. The IIIAB diagram is redrawn from ref. 40. The model for IVA has been slightly improved from ref. 43 to derive updated S and P contents. The gray dots are data from INAA. The red, blue, and green dashed lines represent solid from SFC, solid from trapped melt (TM), and liquid, respectively. The red circles mark the crystallization percentage of a core. The purple crosses are the equilibrium mixing of solid from SFC and solid from trapped melt. Each cross represents a 5% increment.

Elton (officially designated as ungrouped) is reclassified here as IC based on our data. We use 15 wt.% S and 0.49 wt.% P as the optimal composition to fit the interelement trends. Fig. 1*A* shows the result for Ir versus As. Iridium and As are chosen because the large fractionation of Ir during fractional crystallization is diagnostic for distinguishing different S contents (34). Arsenic is determined with high relative precision during analysis (39), and its partitioning behavior is well parameterized (50). Other interelement results are shown in *SI Appendix*, Fig. S1. All interelement trends can be reasonably fitted by 15 ± 2 wt.% S and 0.46 ± 0.03 wt.% P, except for some slight scatter in Mo and Pd (*SI Appendix*, Fig. S1). Our S contents are slightly lower and P contents much higher than the previous estimate of 19 wt.% S and 0.1 wt.% P (49).

The current collection of IC irons represents <26% crystallization of the core. The core has a relatively high trapped-melt amount of ~60%. The formation of trapped melt occurred only at the very beginning and at the late stages of crystallization. The most evolved irons are products of sequential equilibrium mixing (0 to 60%) of simple-fractional-crystallization (SFC) solid and trapped-melt solid.

### Group IIAB.

The interelement trends of Group IIAB can be fitted using bulk 15 wt.% S and 0.5 wt.% P (Fig. 1*B* and *SI Appendix*, Fig. S2). With the bracketing method, the group can be fitted with bulk 15 ± 1 wt.% S and 0.5 ± 0.1 wt.% P. The model can account for all elements (*SI Appendix*, Fig. S2). Our result is consistent with two previous estimates of 17 ± 1.5 wt.% S (34) and 17 wt.% S and 0.7 wt.% P (31), but contrasts with one estimate of 6 wt.% S and 1.5 wt.% P (38).

The IIAB irons represent <30% crystallization of the parent melt. The early crystallized irons (<20%) have small fractions of trapped melt (<15%), and the later-crystallized irons have increasing fractions of trapped melt (up to 60%). The most evolved irons form a continuous mixing line (20 to 60%) of SFC solid and trapped-melt solid.

### Group IIIAB.

The group was recently modeled (40), and we added two elements (Ru and Pd) (31) to the model using the previous determinations of 9 ± 1 wt.% S and 0.32 ± 0.02 wt.% P (40). Both Ru and Pd can be reasonably well fitted (*SI Appendix*, Fig. S3). The modeling of the Ir-As trend is shown in Fig. 1*C*.

### Group IIIE.

We used 7 wt.% S and 0.5 wt.% P as the optimal composition to fit the interelement trends (Fig. 1*D* and *SI Appendix*, Fig. S4). The trends can be well fitted using 7 ± 2 wt.% S and 0.48 ± 0.02 wt.% P. The current collection of IIIE irons represents ~62% crystallization of the core. The core has a relatively low amount of trapped melt (<25%) compared to other NC cores.

### Group IVA.

IVA irons have recently been modeled using 2.9 wt.% S (43). In that model, other than starting from 0% crystallization, it is assumed the initial 40% crystallization products are missing from the current IVA collection. We made a slight improvement to the previous model with bracketed S (3 ± 1 wt.%) and P (0.11 ± 0.01 wt.% P) contents, and added Os, Ru, Mo, Pd, and Rh (42) to the model (*SI Appendix*, Fig. S5), which are reasonably well fitted. Fig. 1*E* shows the modeling for the Ir-As trend.

### Bulk Compositions of the NC-Iron Cores.

**Fig. 2.**
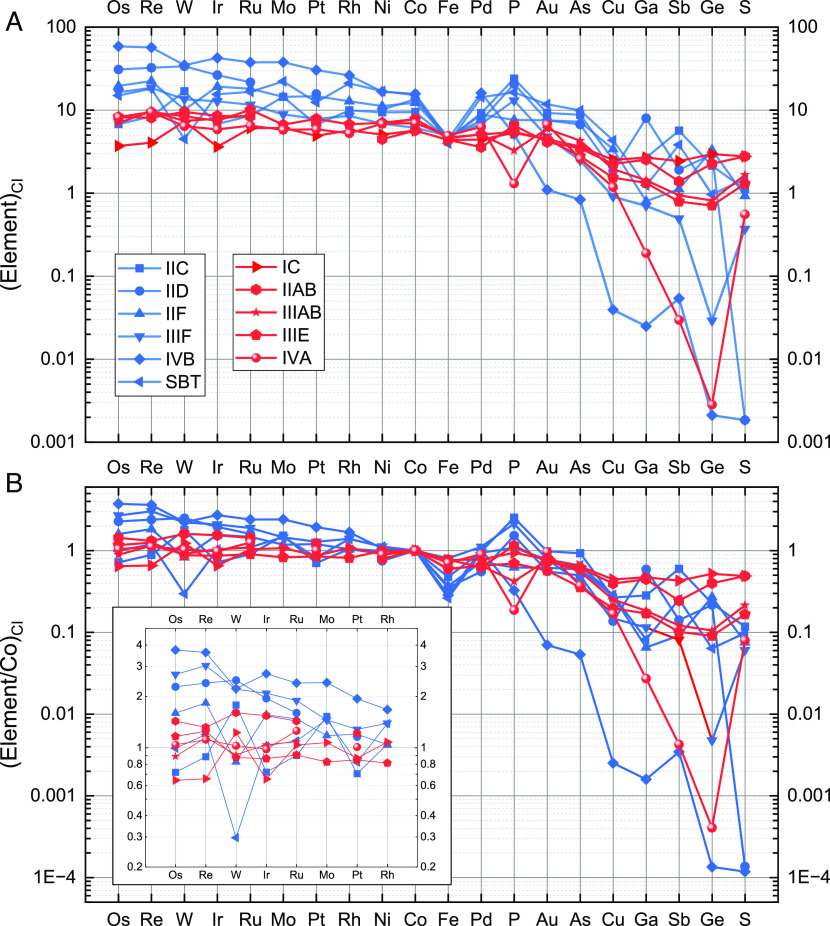
Bulk siderophile abundances of asteroidal cores. (*A*) Siderophile concentrations normalized to CI chondrites (52). (*B*) Siderophile concentrations normalized to Co and CI chondrites. The small panel in (*B*) shows a magnified plot of Os, Re, W, Ir, Ru, Mo, Pt, and Rh. Data of groups IIC, IID, IIF, IVB, and the SBT from ref. 44, IIIAB from ref. 40 with addition of Ru and Pd from this work, IIIF from ref. 51, and IVA from ref. 43 with addition of Os, Ru, Mo, Pd, and Rh from this work. Siderophile elements are ordered by decreasing 50% condensation temperature (T_50_) (53) from *Left* to *Right*, except that we used a relative T_50_ estimate for Au (*SI Appendix*).

Table 1 summarizes the S and P contents derived from this study and our previous studies. Fig. 2 shows the bulk compositions for all NC-iron cores determined from our modeling, plotted in comparison to the previously determined CC-iron core compositions (44). The bulk compositions for all NC cores are given in *SI Appendix*, Table S4 and all CC cores (44, 51) in *SI Appendix*, Table S5. These results and the individual modeling results for each group form the basis for the discussion in the next section.

**Table 1.** Model-derived bulk S and P contents and modal CAI abundances of precursor materials of iron-meteorite groups and the South Byron Trio (SBT)

Group/Grouplet	S (wt.%)	P (wt.%)	CAI in precursor (wt.%)^†^	Reference^*^
NC-type				
IC	15 ± 2	0.46 ± 0.03	0	This study
IIAB	15 ± 1	0.5 ± 0.1	8 ± 9	This study
IIIAB	9 ± 1	0.32 ± 0.02	0	(40), (43) and this study
IIIE	7 ± 2	0.48 ± 0.02	0	This study
IVA	3 ± 1	0.11 ± 0.01	0	(43) and this study
CC-type				
IIC	6 ± 2	2.2 ± 0.3	0	(44) and this study
IID	0.5 ± 0.5	1.9 ± 0.1	15 ± 11	(44) and this study
IIF	5 ± 1	0.65 ± 0.05	9 ± 9	(44) and this study
IIIF	2	1.3	17 ± 12	(51) and this study
IVB	0.5 ± 0.5	0.45 ± 0.02	26 ± 14	(44) and this study
SBT	8 ± 2	1.5 ± 0.3	0	(44) and this study

^*^The bulk S and P concentrations are from the literature, except that the bracketed values of IVA are from this study. All modal CAI abundances are from this study.

^†^The estimated CAI abundances are from Model 2 (a Monte Carlo linear regression model) detailed in *SI Appendix*.

## Discussion

### HSE Abundances and Origins in Iron-Meteorite Cores.

As shown in Fig. 2*A*, the NC-iron cores (red lines) generally have lower CI-normalized HSE abundances (IC: 4×, IIAB: 8×, IIIAB: 7×, IVA: 7×) than CC-iron cores (blue lines) (IIC: 7×, IID: 30×, IIF: 20×, IIIF: 18×, IVB: 55×, SBT: 15×). In CC-iron parent bodies, a high abundance of Fe remains in the silicate portion of the bodies as FeO, resulting in higher relative concentrations of siderophile elements in the cores. To account for the different oxidation states of the parent bodies, previous studies (31, 33, 44) used Ni-normalized bulk chemical compositions to examine relative HSE abundances. However, such normalization may be biased due to the occurrence of schreibersite [(Fe,Ni)_3_P] inclusions in iron meteorites. Coarse schreibersite inclusions can be a significant source of Ni in iron meteorites but are not included in our analyses at UCLA. We analyze only the metal fraction of iron meteorites, while coarse-grained inclusions, including schreibersite, are intentionally avoided. For example, some IIAB irons and all IIG irons are enriched in schreibersite (54), and our INAA (instrumental neutron activation analysis) data show these two groups have the lowest Ni contents among all iron-meteorite groups (20, 38). Therefore, analyses of the metal fraction alone of these irons could lower the estimate of bulk Ni in the IIAB+IIG core. Consequently, in Fig. 2*B*, we choose to normalize all elements to Co and to CI chondrites to minimize the possible overestimate of siderophile abundances due to the exclusion of Ni-bearing accessory minerals in our analyses. Like Ni, Co tends to stay in the core during core–mantle differentiation; unlike Ni, Co is not concentrated in common accessory minerals in iron meteorites.

After normalization to Co and to CI chondrites, the HSE abundances of NC-iron cores (IC: 1×, IIAB: 1.5×, IIIAB: 1×, IVA: 1×) are similar to or lower than those of CC-iron cores (IIC: 0.8×; IID: >2×; IIF: >1.5×; IIIF: >2×, IVB: >3×; SBT: 1×) (Fig. 2*B*). The generally higher HSE abundances of CC-iron parent bodies have been attributed to high HSE abundances in their precursor materials (44). The chondritic HSE abundances in the NC-iron cores (except for Group IIAB) indicate the HSE abundances of their precursor materials resemble those of chondrites.

Group IIAB has higher-than-chondritic HSE abundances and shows a flat, unfractionated pattern on a diagram of HSE abundance versus T_50_ (50% condensation temperature) (Fig. 2*B*). The pattern contrasts with the CC-iron cores with the highest HSE abundances [groups IVB (33, 44) and IID (44)], which show down-sloping HSE patterns toward elements of higher volatility on the HSE abundance versus T_50_ diagram. These abundance patterns, hereafter referred to as “sloped HSE abundance patterns,” provide insight into the source of the HSEs.

CAIs are the main carrier of HSEs in chondrites (44, 55). NC chondrites (enstatite, ordinary, and R groups) have extremely low CAI abundances (0.01 to 0.04 vol.%) (56), and their HSE abundances are close to or lower than that of CI chondrites (57). In contrast, CC chondrites have the same or higher HSE abundances compared to CI chondrites. Carbonaceous chondrites contain various abundances of CAIs (CI, 0 vol.%; CR, 0.6 vol.%; CO, 1.0 vol.%; CM, 1.2 vol. %; CV, 3.0 vol.%; CK, 4.0 vol.%) (56), and these CAI abundances are linearly related to their HSE abundances (44). In some CAIs, HSEs are highly concentrated in refractory metal nuggets (RMNs) (58). HSEs in some RMNs condensed early at high and various temperatures in the solar nebula (53), resulting in, on average, mildly sloped HSE abundance patterns (33, 58). This explains the observation that CAI-rich CV and CK chondrites have elevated HSE abundances and sloped HSE abundance patterns (57). Similarly, the co-occurrence of elevated HSE abundances and sloped HSE abundance patterns in groups IVB and IID suggest the main source of HSEs in their precursor materials is the suite of RMNs formed at high temperatures (44, 56).

In some other CAIs, HSEs are concentrated in Fremdlinge—opaque assemblages consisting of refractory metal alloys (Ru, Rh, Pd, Os, Ir, Pt, Re, W, and Mo), Fe-Ni alloys, oxides, and sulfides. These inclusions were formed during whole-rock aqueous alteration of RMNs (59, 60). A small number of Fremdlinge have a sloped HSE abundance pattern, indicating that the refractory metals within the RMN precursors of Fremdlinge condensed at high and somewhat variable temperatures (61). The majority of Fremdlinge have a flat (unfractionated) HSE abundance pattern (61) that is unlikely to have been produced by aqueous alteration; they retain the primitive signature of the RMN precursors of the Fremdlinge. In this case, the phases within these RMNs condensed at relatively low and similar temperatures, mostly between 1,468 and 1,480 K (61). Group IIAB has elevated HSE abundances, forming a flat abundance pattern (Fig. 2*B*) resembling those in most Fremdlinge with unfractionated HSEs. Such Fremdlinge inclusions are likely enriched in the precursor materials of Group IIAB. Groups IIF and IIIF have intermediately sloped HSE patterns between those of groups IIAB and IVB (Fig. 2*B*). This implies that the IIF and IIIF precursor materials had a mixture of RMNs formed at both relatively low and high temperatures in the solar nebula.

### Constraints on the Structure and Evolution Models of the Disk.

CAIs are the first solids formed in the Solar System (62). According to the CAI abundances in chondrites (56), the region (in the inner disk) where ordinary chondrites (CAI abundance = 0.03 to 0.06 wt.%) accreted had very low CAI abundances, whereas the region (in the outer disk) where carbonaceous chondrites (CAI abundance = 0.8 to 5.6 wt.%, excluding CI) accreted had relatively high, albeit variable, CAI abundances.

**Fig. 3.**
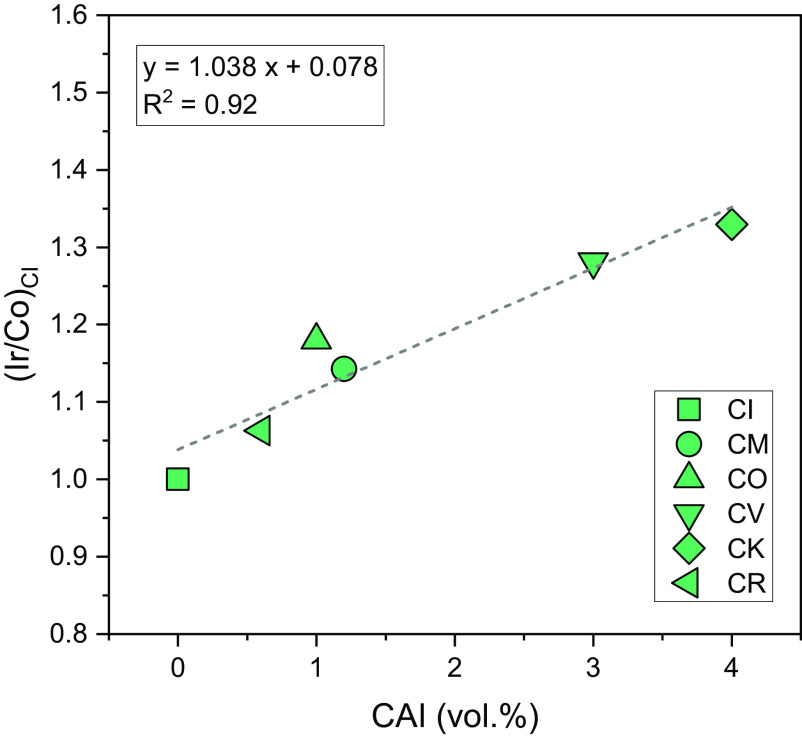
Linear fitting between Ir abundance and CAI abundance (percentage by volume) in carbonaceous chondrites. (Ir/Co)_CI_ denotes Ir concentrations normalized to Co and CI chondrites. Cobalt and Ir concentrations (57, 65, 66) and CAI abundances (56) of carbonaceous chondrites are from the literature.

We use the linear relationship between HSE abundance and CAI abundance in carbonaceous chondrites (44) to estimate the CAI abundances in the precursor materials of iron-meteorite parent bodies. Instead of using Ni- and CI-normalized HSE abundances as shown in ref. 44, here we use the Co- and CI-normalized abundances (Fig. 3). The methods and calculations are detailed in *SI Appendix*. The results are listed in Table 1. Our updated estimates for the CAI abundances in CC-iron precursor materials vary from 0 to 26 ± 14 wt.%. The numbers are overall consistent with those from the previous study using Ni- and CI-normalized HSE abundances (44). The high CAI abundance for the IVB precursor materials has been observed in a few asteroids (63), and some CV and CK chondrites have been found to have high CAI abundances of 16% by area (64), approaching the values that we model for the groups IID and IIIF. Additionally, the paucity of high-CAI asteroids and chondrites could be attributable to the heating and melting of large CAI-rich bodies due to the decay of ^26^Al (t½ = 717,000 y). Thus, CAI-rich bodies would not be expected to be preserved as chondritic meteorite samples but rather would be expected to form differentiated bodies, such as represented by magmatic iron meteorites.

The very low CAI abundances in most NC-iron precursor materials are consistent with those of ordinary chondrites (56). Group IIAB (discussed below) is an exception. In light of the updated CAI abundances of the CC-iron precursor materials (0 to 26 wt.%), we depict the CAI distribution pattern in the first million years of the protoplanetary disk: The inner disk had very low CAI abundances; the outer disk had higher and variable CAI abundances. This pattern did not change drastically when chondrites accreted 2 to 4 Ma after CAI formation (11, 22, 56). Our estimate pushes back the occurrence of the CAI-distribution heterogeneity in the disk to <1 Ma. This is an important constraint on current disk evolution models.

An effective protoplanetary disk evolution model should explain both the cause of the nucleosynthetic isotopic dichotomy and the CAI storage problem. Recently developed models of a ring-structured protoplanetary disk (12–16) can explain the nucleosynthetic isotopic heterogeneity in the disk, but, thus far, no further modeling has been performed to resolve the CAI storage problem in a ring-structured protoplanetary disk. If the natal disk is separated by several pressure bumps, the structured disk models need to explain how CAIs can be distributed across the disk and why the region beyond the water snowline is especially enriched in CAIs. Prior to the ring-structured disk models, the prevalent models maintained that a) proto-Jupiter effectively separated the CC and NC reservoirs inducing the isotopic dichotomy (5, 7), and b) proto-Jupiter’s pressure bump blocked CAIs in the outer disk from spiraling into the Sun, thus causing the relative enrichment and heterogeneous distribution of CAIs in the CC reservoir (11). The Desch model (11) of proto-Jupiter acting as a barrier can account for both the isotopic dichotomy and the CAI storage problem. However, the Desch model (11) may need to be updated with new constraints from the CAI distribution pattern estimated from iron meteorites. These new constraints are that a) the CAI distribution pattern formed as early as <1 Ma and lasted at least another 3 Ma and b) the maximum CAI modal abundance in the carbonaceous-chondrite-like precursors of iron-meteorite parent bodies in the outer disk may have reached ~26 wt.%. Future disk evolution models, including those for a ring-structured disk, should also take into account the CAI distribution pattern revealed by iron meteorites.

**Fig. 4.**
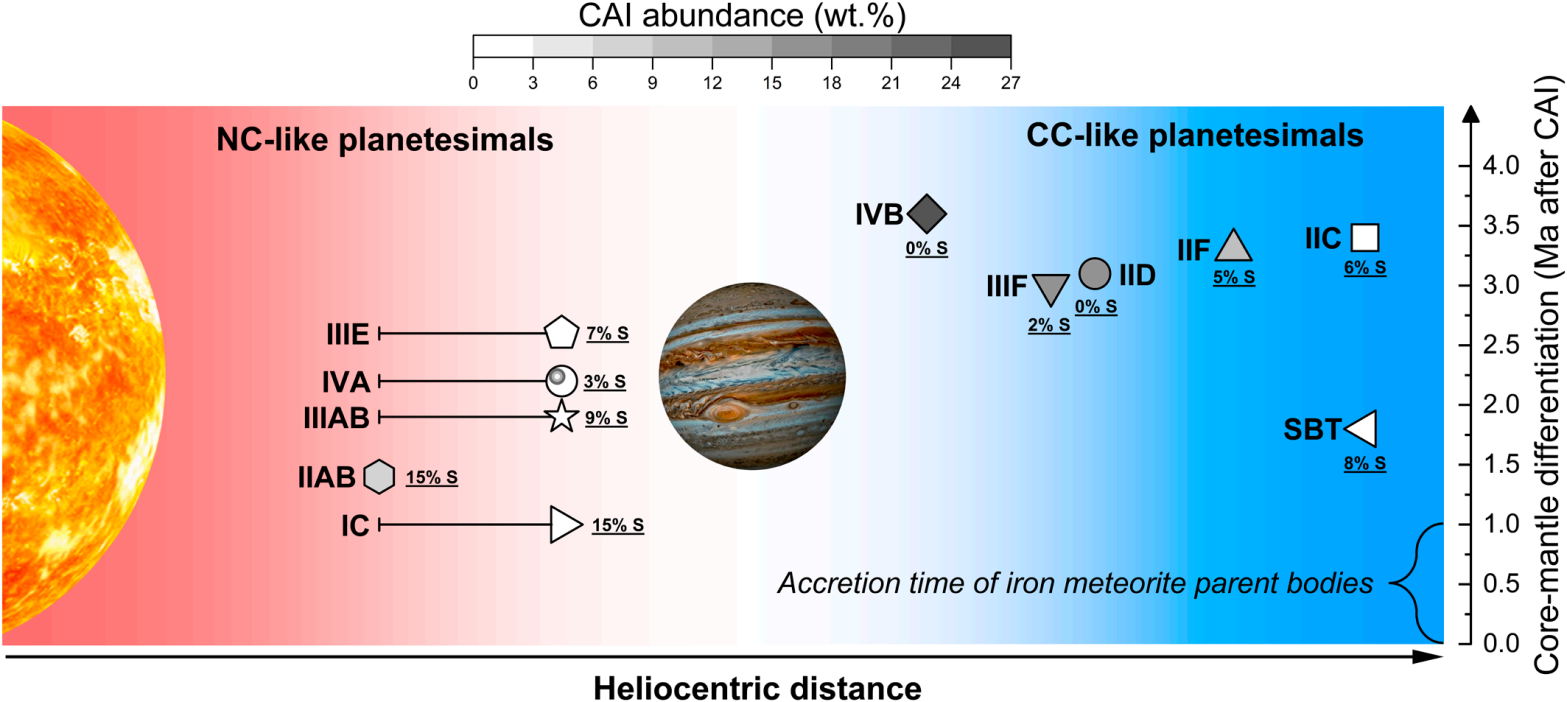
Summary plot of compositions and estimated formation locations of iron-meteorite parent bodies. Each symbol denotes the parent body of an iron group. The positions of the parent bodies on the x-axis (heliocentric distance) show their relative formation locations based on the model-derived CAI abundances of their precursor materials in this study. The error bars of IC, IIIAB, IIIE, and IVA show the range of their possible formation locations, depending on the accretion ages of their parent bodies and the lack of contributions from CAIs. The positions of the parent bodies on the y-axis are the core–mantle differentiation ages after CAI formation. The accretion and differentiation ages are from refs. 5 and 17. The numbers alongside the symbols show the bulk S wt.% of iron cores derived from this study and refs. 40, 43, 44, and 51. The gray gradient of the symbols represents the CAI abundances (wt.%) of precursor materials of iron-meteorite parent bodies. All celestial objects are not scaled to their actual sizes.

We provide a summary below of how our estimate for the CAI distribution in the protoplanetary disk fits in with the Desch model (11). CAIs formed close to the Sun (<1 au) (67) and were soon transported both outward to the cooler regions of the disk and inward toward the Sun due to rapid disk expansion (8). In the first 0.5 Ma, CAIs were abundant in the region close to the Sun (<2 au) and decreased with greater heliocentric distance. The high CAI abundance in IIAB precursor materials indicates this group might have accreted very early within this CAI-rich region. Other NC-iron groups may have formed at a different location that was relatively depleted in CAIs, such as the terrestrial planet-forming region (2 to 3 au). This is consistent with the estimate that the IIAB parent body formed in a more reduced condition than other NC-iron parent bodies (68). Alternatively, the vast majority of CAIs may have spiraled into the Sun by the time these other NC iron-meteorite parent bodies formed. The formation of proto-Jupiter separated the disk into the CC and NC reservoirs at ~3.0 au (at 0.6 Ma). In the CC reservoir, proto-Jupiter formed a pressure bump that blocked the infall of CAIs, and vast numbers of CAIs were trapped in this pressure bump (5, 8, 10). The formation of the pressure bump caused the heterogeneous CAI distribution beyond proto-Jupiter with CAI abundance decreasing with greater heliocentric distance (11, 44). This CAI distribution pattern in the outer disk explains the various CAI abundances in both carbonaceous chondrites and the precursor materials of CC-iron parent bodies. Using the CAI abundances of these precursor materials and the meridional distribution pattern of CAIs in the Desch model (11), we estimate the relative formation locations of iron-meteorite parent bodies in the disk (Fig. 4).

### The Behaviors and Distribution Patterns of Volatile and Moderately Volatile Elements.

Taking all CC and NC groups together, we do not see an overall difference in moderately volatile abundances or distribution patterns for siderophile elements (Fig. 2*B*). This agrees with limited differences in their carbon and nitrogen contents (69–71). The volatile abundances and distribution patterns are controlled by the combination of condensation, melting, and crystallization processes. The flat (element/Co)_CI_ patterns formed by As, Cu, Ga, Ge, and S (in order of decreasing T_50_) for IC and IIAB indicate these two cores experienced the least loss and fractionation of volatile and moderately volatile elements. Around 40 to 50% of these volatile elements were lost in the nebular condensation phase, and the flat abundance patterns show these elements may have condensed near or below the T_50_ of S (672 K). This means that volatile and moderately volatile elements in these two iron cores were mostly lost during the condensation of their precursor materials. The later melting and crystallization processes had the least influence on the behaviors and bulk distributions of volatile and moderately volatile elements. For groups IVA and IVB with a steeply sloped pattern of (element/Co)_CI_ versus T_50_ for moderately volatile elements As, Cu, Ga, and Ge (Fig. 2), nebular fractionation processes or devolatilization during the parent-body melting phase likely determined the low abundances and sloped pattern of these elements. Zinc and Cu (moderately volatile) isotopic compositions of groups IVA (72) and IVB (73) do not show signatures of evaporation, so this favors nebular processes such as incomplete condensation. Silver (volatile) isotopic systematics, however, demonstrate that the IVB core might have experienced volatile loss induced by impact disruption, during which the mantle was removed and volatiles in the core were lost without causing large kinetic isotopic fractionation (74). This scenario is consistent with the diverse cooling rates of IVB irons supporting the suggestion that the core cooled without a mantle (75). The bulk abundances and distribution patterns of volatiles and moderate volatiles in other iron groups stay mainly within the envelope of IC+IIAB and IVB in Fig. 2*B*, and these intermediate groups may have had their volatile-element abundances modified by condensation, melting, and crystallization processes.

In our previous study (44), we concluded there might be a difference in bulk P and S abundances between the CC- and NC-iron cores and that the difference was inherited from the chondritic precursor materials of the iron meteorites. The average bulk P concentrations of CC-iron cores are higher than those of NC-iron cores (Table 1 and Fig. 2*A*), but when the P contents are normalized to CI chondrites and Co, the (P/Co)_CI_ values of CC- and NC-iron cores largely overlap (Fig. 2*B*). Our data show that P abundances may not show a significant difference related to the formation locations of iron meteorites. Our estimates of bulk P contents constitute a lower limit due to the limited data and challenges in obtaining accurate bulk measurements of the P contents of iron meteorites. Phosphorus is common in large accessory phases like schreibersite, and consequently, the bulk P data used in this study are from modal analyses (54). The scattered P abundances of all groups compared with the adjacent Pd and As abundances may have been caused by inaccurate bulk P determinations due to this complication or may imply that melting and crystallization processes also affected the estimates of bulk P (Fig. 2). Some NC-iron cores have higher bulk S concentrations than CC-iron cores (Fig. 2 and Table 1). When the S concentrations are normalized to CI chondrites and Co, the (S/Co)_CI_ values of NC-iron cores (except Group IVA) are higher than those of CC-iron cores. A possible explanation is that a higher proportion of S in the outer disk was oxidized and entered the gas phase before the remaining S was incorporated into the CC-iron cores. Thus, the difference in S abundances between the CC- and NC-iron cores may be related to the formation locations of the parent asteroids. Using the updated bulk S concentrations for NC groups, we further confirm that the bulk S content of a core is correlated with how early its core–mantle differentiation occurred (36, 76). The highest-S parent bodies (IC and IIAB) differentiated earliest, and the lowest-S parent bodies (IVB and IID) differentiated latest among all iron-meteorite groups.

### Crystallization Processes of Asteroidal Cores.

All NC-iron groups sample only a fraction of their metallic cores. Irons from the high-S groups IC and IIAB represent only <30% crystallization products of the cores. Irons from intermediate-S groups (IIIAB and IIIE) sample <60% of the cores. The low-S Group IVA may have sampled up to 80% of the core but the first 40% is not sampled in the current collection (43). These data are comparable to those of the CC-iron cores in which, the higher the S content a core has, the lower crystallization percentage of its corresponding iron group samples (44). This is consistent with S-rich iron-meteorite samples being rare and apparently underrepresented in our meteorite collections (26). Overall, the NC-iron groups have more members than the CC-iron groups (56). The number of members in an iron-meteorite group, to a certain degree, may reflect the size of the core but may also be due to parent-body residency in a location with favorable transport to Earth. Nonetheless, these iron-meteorite parent bodies have relatively small core mass fractions, an average of 21% for NC-iron cores and 13% for CC-iron cores (31), compared with those of terrestrial planets: Mars at 25% (77) and Earth at 32.5% (78). If the iron-meteorite parent bodies are representative of initial accretion materials for terrestrial planets, later accretion of highly reduced materials (such as metal-rich pebbles) is needed to account for the relatively high core mass fractions of Mars and Earth (79).

The amount and formation stage of trapped melt vary among NC-iron cores. The IC core may have produced a significant amount of trapped melt only at the earliest stages, and the later-crystallized irons have much less trapped melt. The IIAB core did not produce a large amount of trapped melt until the latest stages of crystallization. The last few IIAB irons lie on a single mixing line of SFC solid and trapped-melt solid. It seems these irons mark the crystallization of the metallic melt residue in the core. This trapped melt may have occurred at the boundary of immiscible P-rich and S-rich melts that formed as the core crystallized (20). The high schreibersite content in the most evolved IIAB irons and in all IIG irons (20, 38) further supports the notion these irons crystallized from P-rich melts. The IIIAB core has the most abundant trapped melt among all iron-meteorite cores. Some IIIAB and IVA irons crystallized directly from trapped melt, especially at the latest stages of crystallization (Fig. 1 *C* and *E*), and the envelope of the equilibrium mixing of SFC solid and trapped-melt solid was occupied by a large number of IIIAB irons (39, 40). Group IIIE is the only NC core that did not have a large amount of trapped melt throughout the entire crystallization process.

In comparison to the amounts of trapped melt estimated to have occurred in the CC-iron cores (44), the NC-iron cores had larger amounts of trapped melt during their solidification. In other words, the CC-iron cores crystallized from metallic melts with relatively simple crystallization processes, perhaps due to more efficient convection. In contrast, many NC-iron cores may have more complex crystallization structures (such as dendrites, liquid immiscibility, and cracks formed by thermal contraction) or experienced external, impact-induced disturbances that affected effective global-wide convection. Another notable observation is that the initial S content in a core does not play a key role in determining the amount of trapped melt or at which crystallization stage trapped melt will form. The SBT, IIIAB, and IIIE cores have similar initial S contents (8 to 9 wt.%), but only the IIIAB core produced a large amount of trapped melt throughout its crystallization history. The IC and IIAB cores have 6 wt.% more S than the IIIAB core. However, the IC and IIAB cores had a significant amount of trapped melt only at the very early and late stages, respectively, of crystallization. Hence, we conclude the formation of trapped melt in an asteroidal core is not directly related to the initial S content; instead, the internal crystallizing structures and/or external collisional or tectonic disturbances of the core may exert more influence on the amount and timing of trapped melt during core crystallization.

The isotopic (5, 8, 9) and chemical differences between iron groups from the inner and outer protoplanetary disk carry over to the crystallization processes of these groups. Our results show the difference in crystallization process is not controlled by the compositions (such as S contents) of metallic melts. The morphologies and external environments of the cores may be more important factors that diversify the crystallization processes. The morphologies (such as core sizes related to oxidation conditions) and external environments (such as impacts related to disk dynamics) are ultimately determined by stochastic processes and the formation locations of the CC- and NC-iron cores in the disk, as are the isotopic and chemical differences between the two suites.

## Summary

We comprehensively examine the compositions and crystallization processes of all magmatic iron-meteorite groups. We find that the differences in composition, crystallization process, and morphology of asteroidal cores are related to the formation locations of their parent asteroids in the protoplanetary disk. The higher CAI (main carrier of HSEs) abundances in the outer Solar System elevated the siderophile-element abundances in some cores. We reconstruct the meridional CAI distribution across the protoplanetary disk within the first million years of Solar-System history. Our results show that CAIs were depleted in the inner disk and enriched (albeit heterogeneously distributed) in the outer disk. The outer-Solar-System cores have relatively simpler crystallization processes, which may indicate more effective global convection than their inner-Solar-System counterparts. The inner-Solar-System cores might have developed more complex internal structures that affected the crystallization processes. The particularities of these core crystallization structures are likely functions of the chemistry and/or evolution dynamics in the inner Solar System. We conclude that the heterogeneity of chemical attributes and dynamics of the protoplanetary disk formed very early in Solar-System history and determined the diversity of magmatic iron meteorite parent bodies in composition, crystallization process, and morphology. Our previous and present studies of magmatic iron meteorites provide not only constraints on the formation mechanism of planets and planetesimals (especially metallic cores) but also constrain the conditions and processes in the protoplanetary disk that led to planet formation. In particular, future models of the evolution and structure of the disk should account for the pattern and timing of the distribution of CAIs as constrained by our iron-meteorite results.

## Materials and Methods

Mean concentrations of Co, Ni, Cu, Ga, Ge, As, Ru, Sb, Os, Re, Ir, Pt, and Au in groups IC, IIIE, and IIAB were obtained by John Wasson using INAA (instrumental neutron activation analysis) at UCLA over a period of more than 50 y though most of the measurements for groups IC and IIIE were never published in his lifetime; the mean calculations of Ga and Sb include radiochemical neutron activation analysis (RNAA) data in the literature. The INAA method is described in ref. 38. Iron meteorites were sawed to form rectangular specimens with a thickness of 3 mm and a mass of ~550 mg. The specimens were washed with ethanol and then wrapped with aluminum foil. The wrapped specimens were irradiated in the nuclear reactor at the University of California, Irvine. The irradiated specimens were acid-washed with dilute H_2_SO_4_, HCl, and HNO_3_ solutions to remove superficial contamination. Counting started on the same day as the irradiation and was performed four times, after 6, 15, 80, and 600 h, on a hyperpure planar germanium detector over a period of 1 mo. Each batch of INAA samples was monitored by three standard specimens: North Chile [Filomena] (IIAB), Coahuila (IIAB), and NBS steel NBS809B. Counting data were processed by in-house software to generate concentration data. In most cases, each iron meteorite was analyzed twice (two different specimens) to calculate the mean concentrations. Analyses made after 1986 were given 1.5 to 2× weight in the mean calculations. Except for the irradiation, all pretreatment, counting, and data processing were performed at UCLA.

The concentrations of 14 elements in IC, IIAB, and IIIE irons are listed in *SI Appendix*, Table S2. INAA replicates of each sample are listed in *SI Appendix*, Table S3. The relative 95% confidence limits on the mean concentrations in *SI Appendix*, Table S2 are 1.5 to 3% for Co, Ni, Ga, Ir (concentrations >0.1 µg/g), and Au; 4 to 6% for As, Ge (by RNAA), and Sb; 7 to 10% for W (values >0.3 µg/g), Re (>50 ng/g), Ru (>4 µg/g), and Pt (>2 µg/g). The means of Cr have confidence limits at >10% because Cr in iron meteorites is present mainly as chromite and daubréelite. The ^54^Fe(n,α)^51^Cr fast-neutron reaction also causes interference in the determination of Cr, and the degree of interference is about 6 μg Cr per gram of Fe (41). For these reasons, we do not include Cr in the fractional crystallization modeling. The P data used in this study are from modal analyses of iron meteorites (54).

We use an updated fractional crystallization modeling method (40) and partitioning parameterizations (50) to simulate the crystallization processes of the target asteroidal cores. Our INAAs include Cr, Co, Ni, Cu, Ga, Ge, As, Ru, Sb, W, Re, Os, Ir, Pt, and Au. We also use Mo and Rh by previous LA-ICP-MS (laser ablation inductively coupled plasma mass spectrometry) analyses and more-precisely determined Ru, Re, Os, Pt, and Pd by isotope dilution (ID-) ICP-MS (31, 40, 42, 48, 49). Data sources of each element used in the models can be found in *SI Appendix*, Table S2. The detailed modeling methods are described in *SI Appendix*.

## Supplementary Material

Appendix 01 (PDF)

Code S01 (TXT)

## Data Availability

All other data are included in the manuscript and/or supporting information, and all INAA data will be made available in The UCLA Cosmochemistry Database (https://www.astromat.org/collections/ucla-cosmochemistry-database/) (80).
