# Supplementary material for: Compositions of iron-meteorite parent bodies constrain the structure of the protoplanetary disk
Source: Proc Natl Acad Sci U S A. 2024 May 28;121(23):e2306995121. doi: 10.1073/pnas.2306995121 (PMC11161762; doi:10.1073/pnas.2306995121)
Supplement: Supplementary file 1 — Appendix 01 (PDF) [file pnas.2306995121.sapp.pdf]

PNAS

Supporting Information for

Compositions of iron-meteorite parent bodies constrain the structure of the protoplanetary disk

Bidong Zhang, Nancy L. Chabot, Alan E. Rubin

Corresponding author: Bidong Zhang
Email: bdzhang@ucla.edu

This PDF file includes:

Supporting Text
Figures S1 to S7
Tables S1 to S5
SI References

Supporting Text

Modeling methods

Siderophile elements in magmatic iron meteorites show these meteorites formed by fractional crystallization of metallic melts. Sulfur and P concentrations in a metallic core increase as crystallization proceeds and affect the behaviors of other siderophile elements. Partition coefficients of siderophile elements at different S and P concentrations have been quantified by experiment-derived parameterizations (1). In iron meteorites, both P and S concentrate in phases other than Fe-Ni metal; to determine accurate bulk compositions, these other phases must also be measured. Estimates of P contents in iron meteorites have been determined through modal abundances that account for schreibersite. During core crystallization, S forms troilite (FeS); however, because most iron meteorites lack large troilite inclusions, similar estimates of the modal abundance of S are not possible. Hence, S contents in irons cannot be directly measured and have instead been determined by modeling metal crystallization trends (1-4). The bulk S content of a metallic core can be determined by iterative modeling of interelement trends of siderophiles (5, 6). Due to the variability of partition coefficients of siderophiles throughout the crystallization process, we use small-step batch crystallization to simulate fractional crystallization. Another assumption in such fractional crystallization modeling is that concentrations of siderophile elements in irons are the result of equilibrium mixing of crystallized solid metal and solids crystallized from trapped melt (7). By fitting the interelement trends of siderophile elements for an iron-meteorite group, one can determine the bulk concentrations of these elements in the metallic core. The fractional-crystallization modeling methods are described in detail in the literature (5, 7). We provide a short summary below.

Equilibrium batch crystallization is a simple mass balance between the phase fields:

$$\frac{C_L}{C_i} = \frac{1}{(1 - f + f \times D_E)} \tag{1}$$

In Eq. 1, C_i, C_L, f, and D_E represent the bulk composition of the liquid, the bulk composition of the remaining liquid, the crystallization step, and the partition coefficient of an element, respectively. The models in this study used a constant of 0.001 for each mass step. The concentration of an element in the solid (C_S) derived from each mass step is calculated using the bulk composition of the remaining liquid and the partition coefficient of the element in that step:

$$C_s = D_E \times C_L \tag{2}$$

The partition coefficient of an element is strongly influenced by the S and P contents of the liquid and varies at each small step. D_E is parameterized using Eq. 3 (5).

$$D_E = D_0 \times (Fe\ domains)^{\beta} \tag{3}$$

D_0 is the partition coefficient of an element in the S- and P-free system. β is a constant specific to an element related to its behavior in a S or P-bearing liquid. *Fe domains* represent the fraction of free Fe atoms available in the liquid (8). *Fe domains* in the Fe-Ni-S-P system were calculated using Eq. 4, and β_{S+P} of an element in the Fe-Ni-S-P system was calculated using Eq. 5 (9).

$$Fe\ domains = \frac{1 - 2X_S - 4X_P}{1 - X_S - 3X_P} \tag{4}$$

$$\beta_{S+P} = \left[\frac{2X_S}{(2X_S + 4X_P)}\right]\beta_S + \left[\frac{4X_P}{(2X_S + 4X_P)}\right]\beta_P \tag{5}$$

X_S and X_P are the molar fractions of S and P in the liquid, respectively. β_S and β_P are the beta values for each element in the Fe-Ni-S and Fe-Ni-P systems, respectively.

The scattered interelement trends of Group IIIAB were originally proposed to be caused by the equilibrium mixing of solid and liquid (trapped melt) (6), which is called the trapped-melt model. A recently revised version of the trapped-melt model considers the formation of troilite in the trapped melt (7). The relationship between trapped melt ($C_{Trapped\ melt}$) and the solid ($C_{Trapped\ melt\ solid}$) that crystallized from the trapped melt can be expressed using Eq. 6:

$$C_{Trapped\ melt\ solid} = \frac{C_{Trapped\ melt}}{1-x} \quad (6)$$

where x denotes the mass fraction of trapped melt that solidifies to form troilite. In this study, we consider the formation of all groups and evaluate the fraction of trapped melt for each group.

Concentrations of Cr, Co, Ni, Cu, Ga, Ge, As, Sb, Ru, Re, Os, W, Ir, and Au are determined by NAA. Some Ru and Os data and all Rh, Pd, and Mo data are from ICP-MS data in the literature. Phosphorus concentrations are from modal analyses (10). Details of the data sources are shown in Table S1. All elements are plotted against As. In a metallic core, this element varies by a factor of four or more, and it can be determined by INAA at relatively low uncertainties (4–6%). The use of As is an updated practice from Au in fractional crystallization modeling. Arsenic and Au have almost identical behaviors during fractional crystallization, but the partition coefficient of Au is relatively poorly understood in low-S melts (7, 8). We therefore use element-versus-As trends in the modeling.

The bulk compositions of siderophiles in each core are determined by the trial-and-error method. We assume the lower boundary of the envelope of an element-As trend overlaps with the SFC solid track. The bulk elemental concentrations (especially S and P) are adjusted to fit as many element-versus-As trends as possible at the same time. An optimal initial S content is thereby obtained. Increments of ±1% S around the optimal S value are used to achieve similar fitting of models under the optimal S value, and this bracketing method allows us to take the analytical and modeling uncertainties into consideration. The modeling results using optimal S and P contents for each group are shown in Figs. S1-5. For group IIIAB, we show only our new modeling results for the elements Ru and Pd, as the other elements were modeled in Ref (7). For group IVA, we show our new modeling results for P, Mo, Ru, Rh, Pd, and Os, as the other elements were modeled in (29).

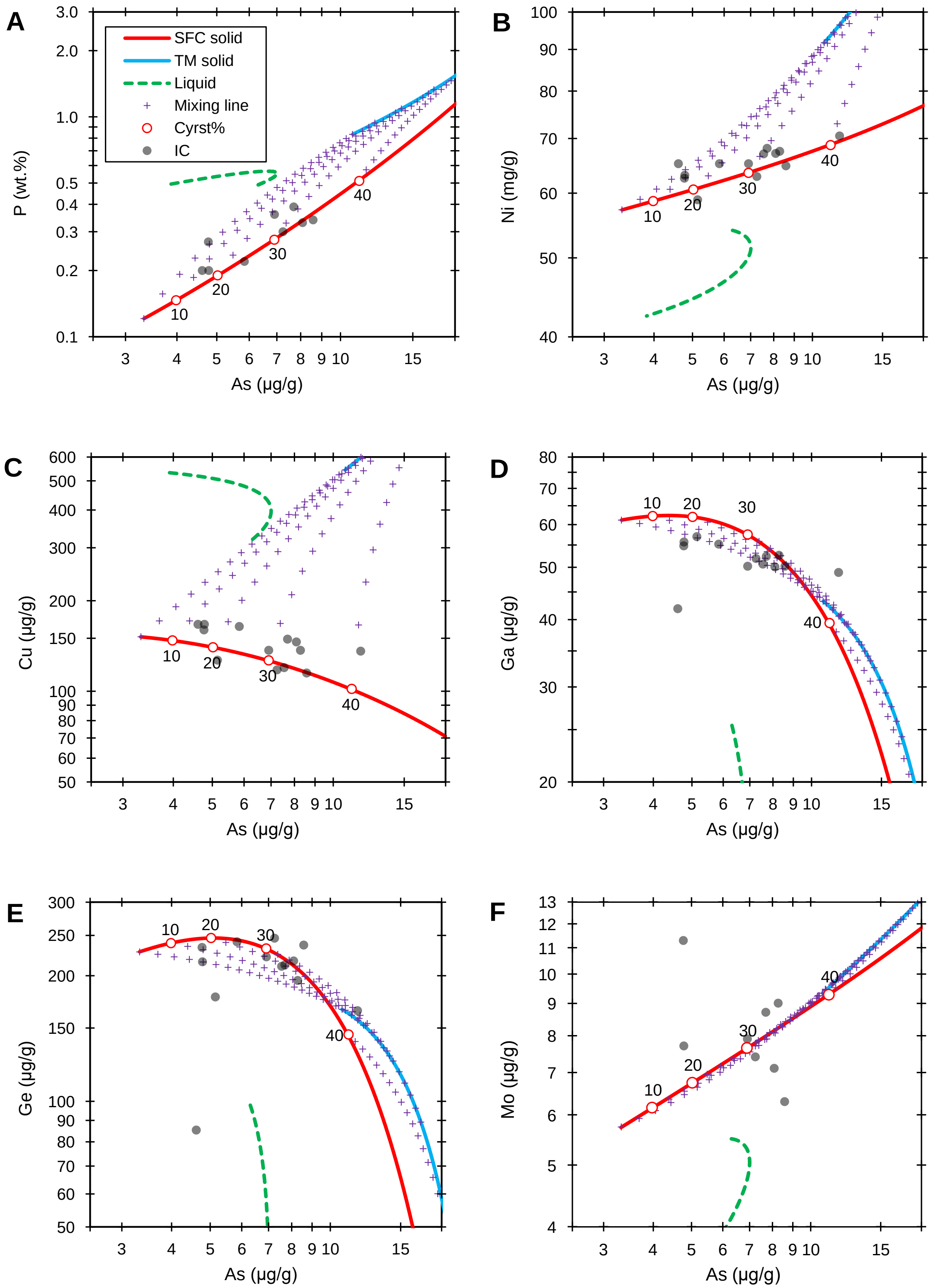

A
SFC solid
TM solid
Liquid
Mixing line
Cyrst%
IC
P (wt.%)
As (μg/g)
B
Ni (mg/g)
As (μg/g)
C
Cu (μg/g)
As (μg/g)
D
Ga (μg/g)
As (μg/g)
E
Ge (μg/g)
As (μg/g)
F
Mo (μg/g)
As (μg/g)

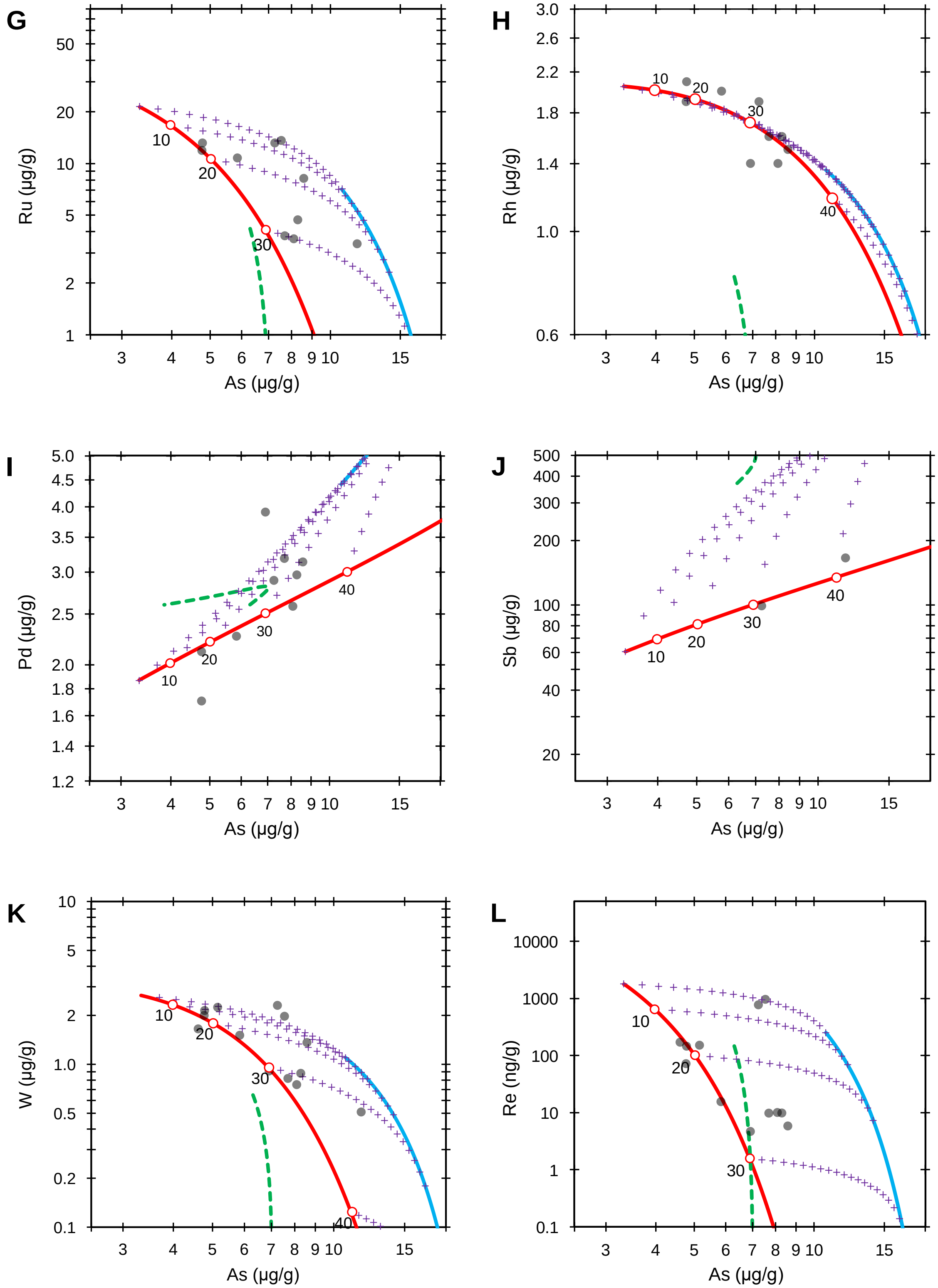

G
Ru (µg/g)
As (µg/g)
H
Rh (µg/g)
As (µg/g)
I
Pd (µg/g)
As (µg/g)
J
Sb (µg/g)
As (µg/g)
K
W (µg/g)
As (µg/g)
L
Re (ng/g)
As (µg/g)

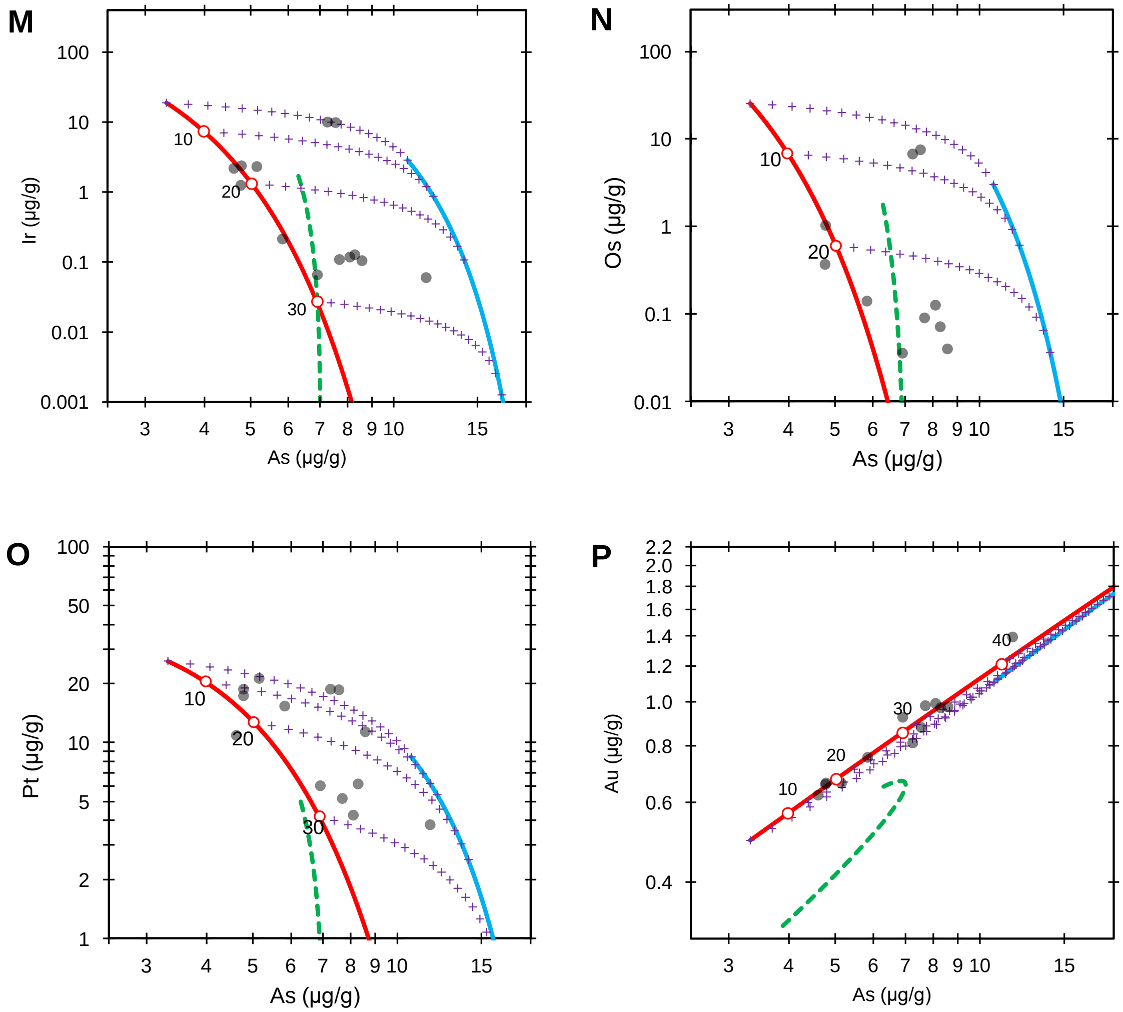

Figure S1. Fractional crystallization modeling of element-versus-As trends in group IC. The figures show results from the optimal bulk 15 wt.% S and 0.46 wt.% P. The black dots are NAA or ICPMS data. The red, blue, and green dashed lines are the solid from simple fractional crystallization (*SFC solid*), solid from trapped melt (*TM solid*), and liquid (*Liquid*), respectively. The purple crosses are the equilibrium mixing (*Mixing line*) between SFC and trapped-melt solids at an increment of 5%. The labeled circles on the red lines represent the crystallization sequence (*Cryst%*).

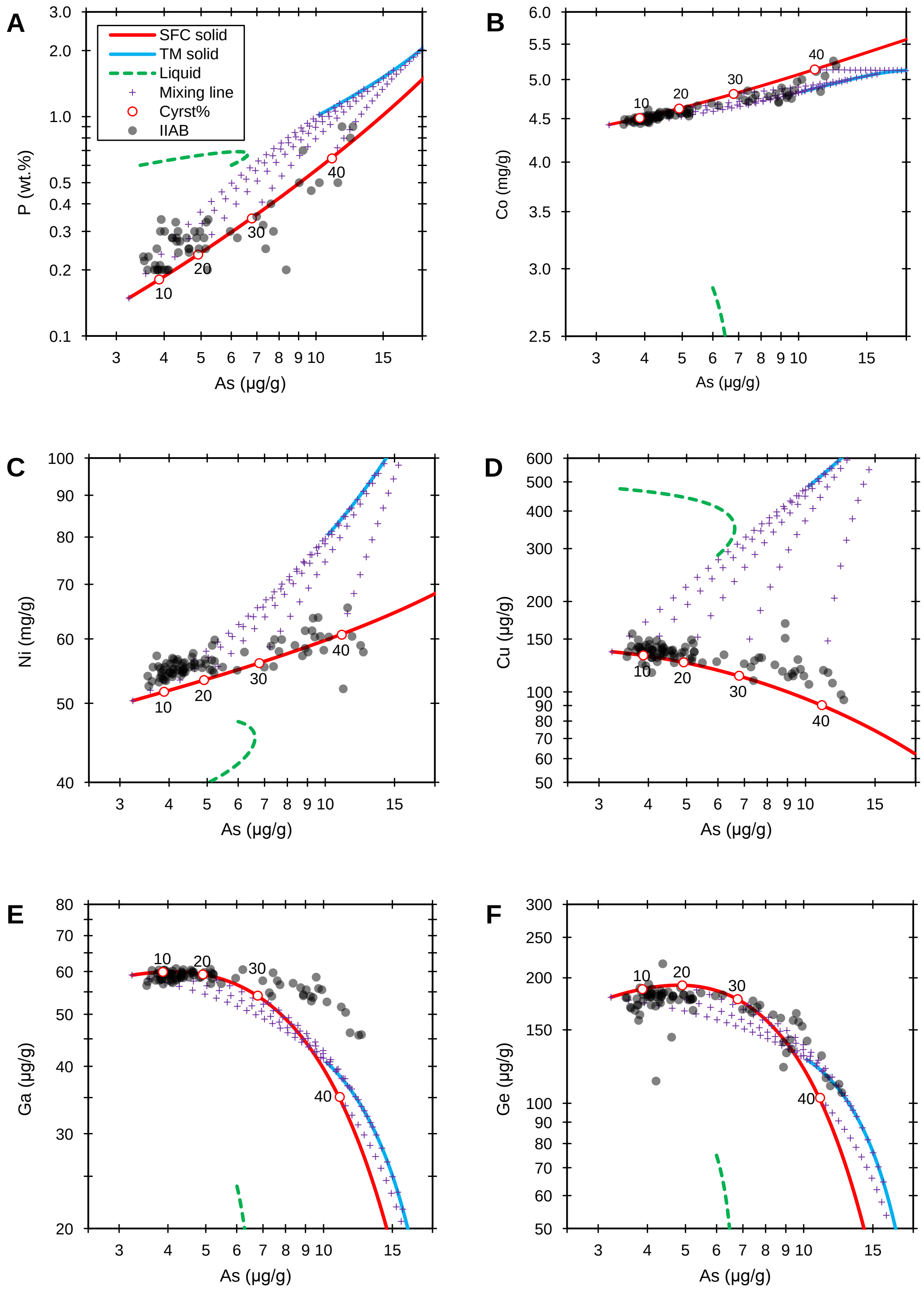
A
SFC solid
TM solid
Liquid
Mixing line
Cyrst%
IIAB
P (wt.%)
As (µg/g)
B
Co (mg/g)
C
Ni (mg/g)
D
Cu (µg/g)
E
Ga (µg/g)
F
Ge (µg/g)

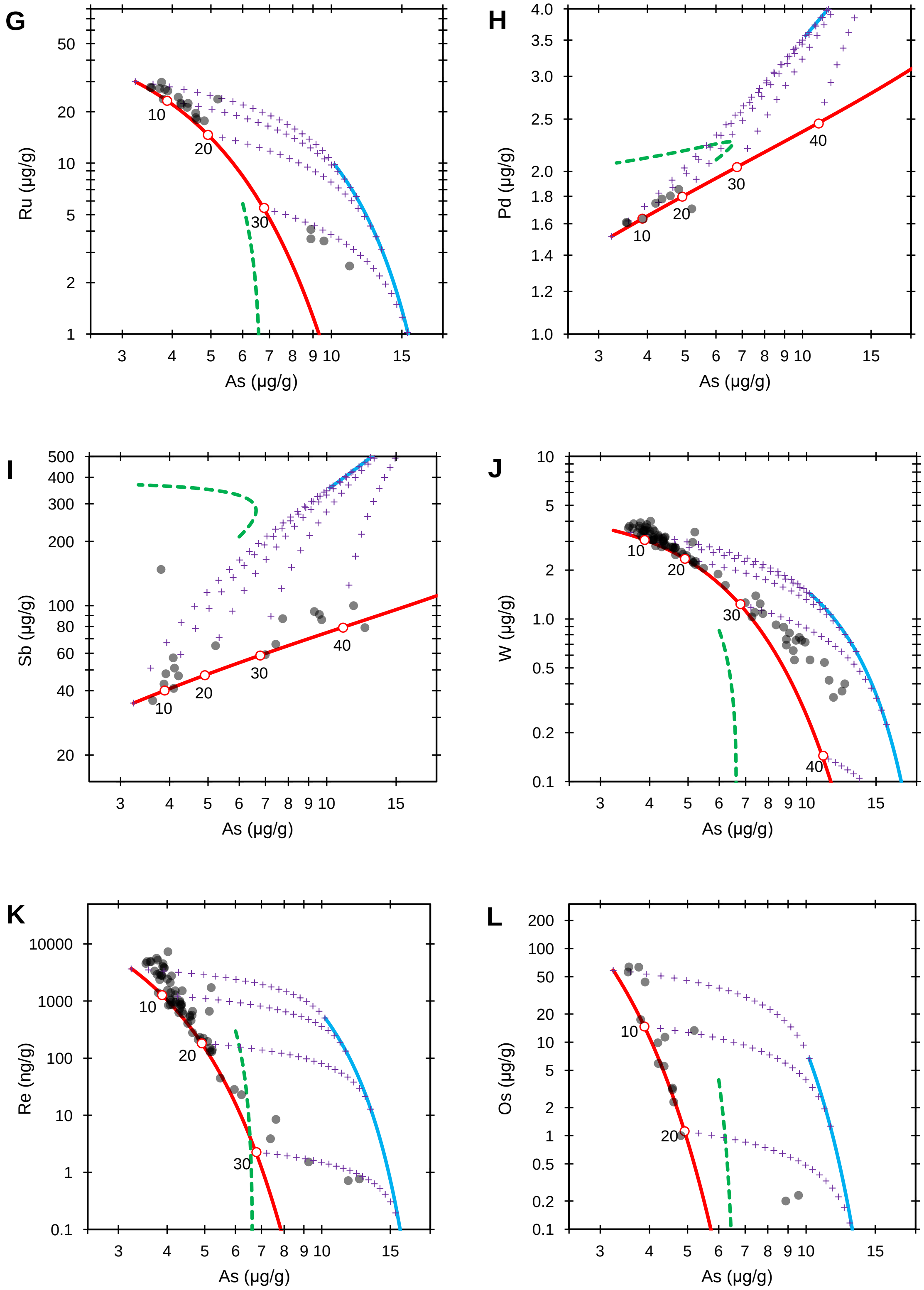
G
Ru (µg/g)
As (µg/g)
10
20
30
H
Pd (µg/g)
As (µg/g)
10
20
30
40
I
Sb (µg/g)
As (µg/g)
10
20
30
40
J
W (µg/g)
As (µg/g)
10
20
30
40
K
Re (ng/g)
As (µg/g)
10
20
30
L
Os (µg/g)
As (µg/g)
10
20

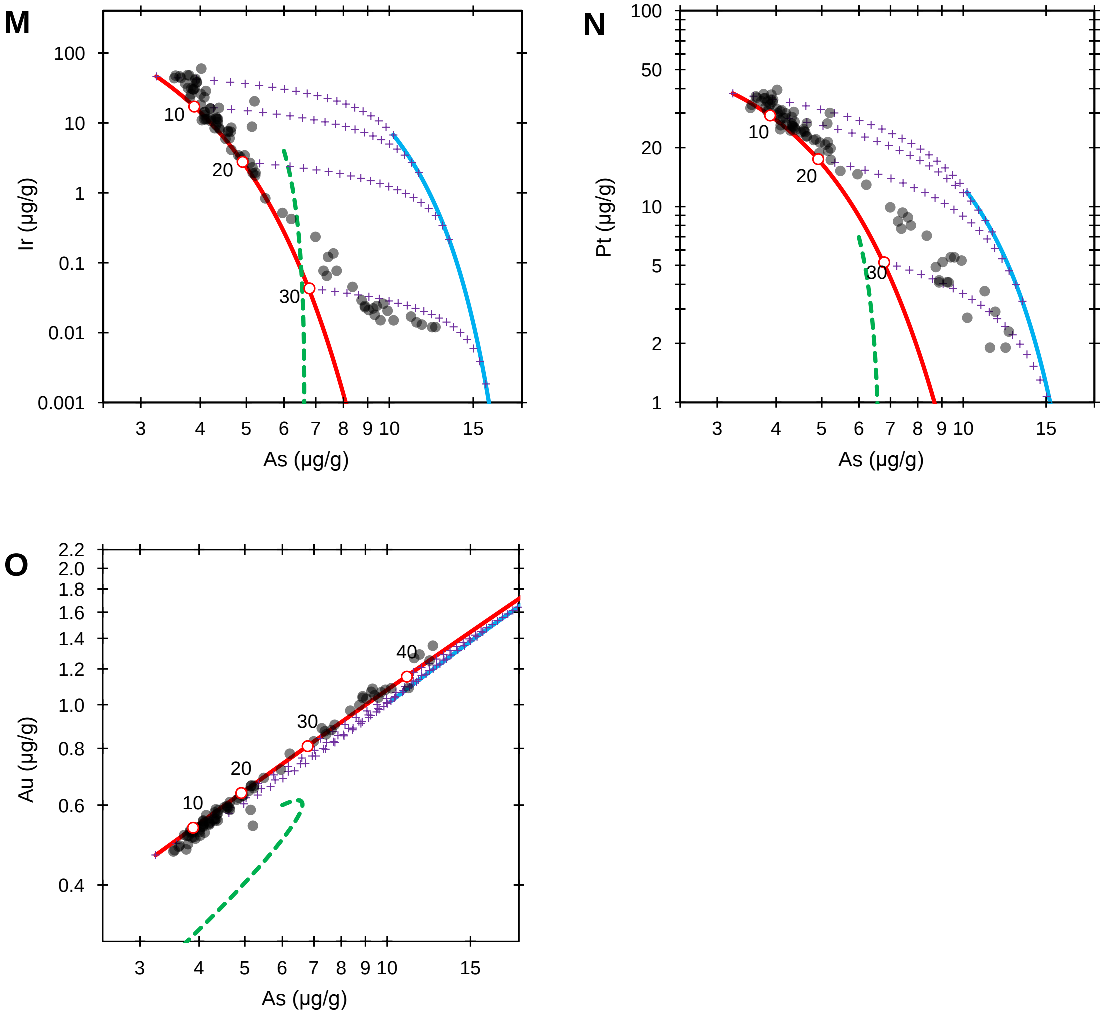

Figure S2. Fractional crystallization modeling of element-versus-As trends in group IIAB. The figures show results from the optimal bulk 15 wt.% S and 0.50 wt.% P. The black dots are NAA or ICPMS data. The red, blue, and green dashed lines are the solid from simple fractional crystallization (*SFC solid*), solid from trapped melt (*TM solid*), and liquid (*Liquid*), respectively. The purple crosses are the equilibrium mixing (*Mixing line*) between SFC and trapped-melt solids at an increment of 5%. The labeled circles on the red lines represent the crystallization sequence (*Cryst%*).

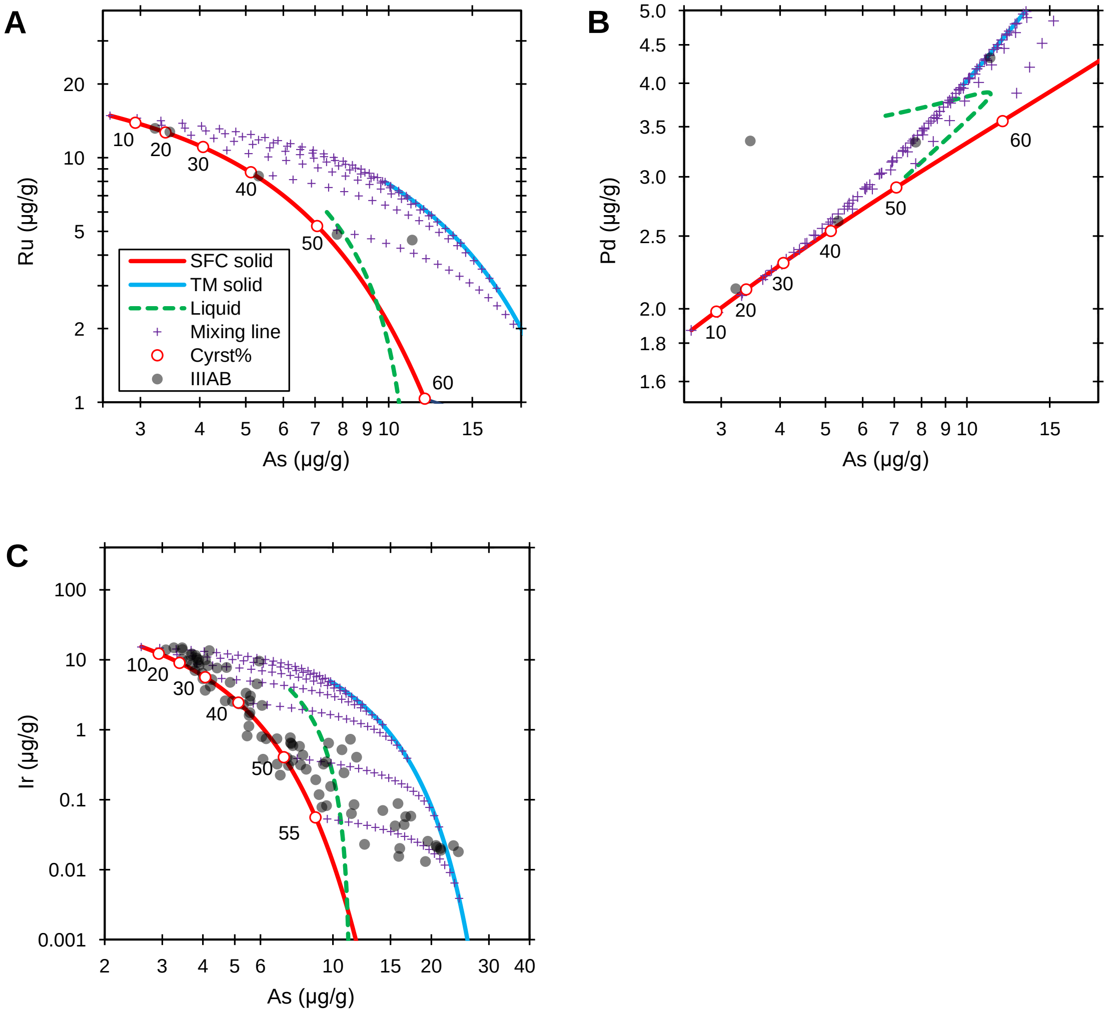

Figure S3. Fractional crystallization modeling of elements (Ru, Pd, and Ir) versus As trends in group IIIAB. The figures show results from the optimal bulk 9 wt.% S and 0.32 wt.% P. The black dots are ICPMS data. The red, blue, and green dashed lines are the solid from simple fractional crystallization (*SFC solid*), solid from trapped melt (*TM solid*), and liquid (*Liquid*), respectively. The purple crosses are the equilibrium mixing (*Mixing line*) between SFC and trapped-melt solids at an increment of 5%. The labeled circles on the red lines represent the crystallization sequence (*Cryst%*).

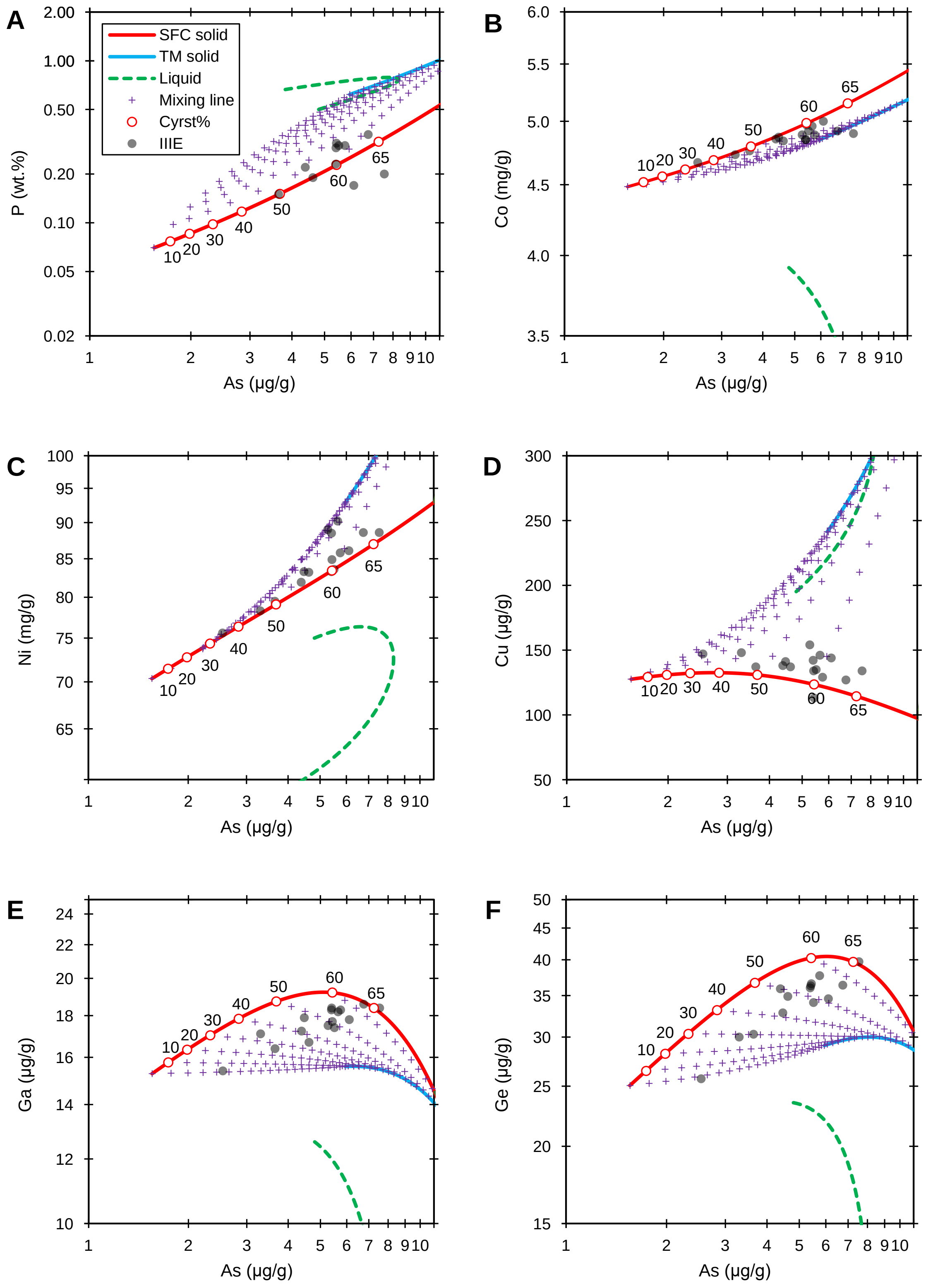

A
B
C
D
E
F
SFC solid
TM solid
Liquid
Mixing line
Cyrst%
IIIE
P (wt.%)
Co (mg/g)
Ni (mg/g)
Cu (µg/g)
Ga (µg/g)
Ge (µg/g)
As (µg/g)

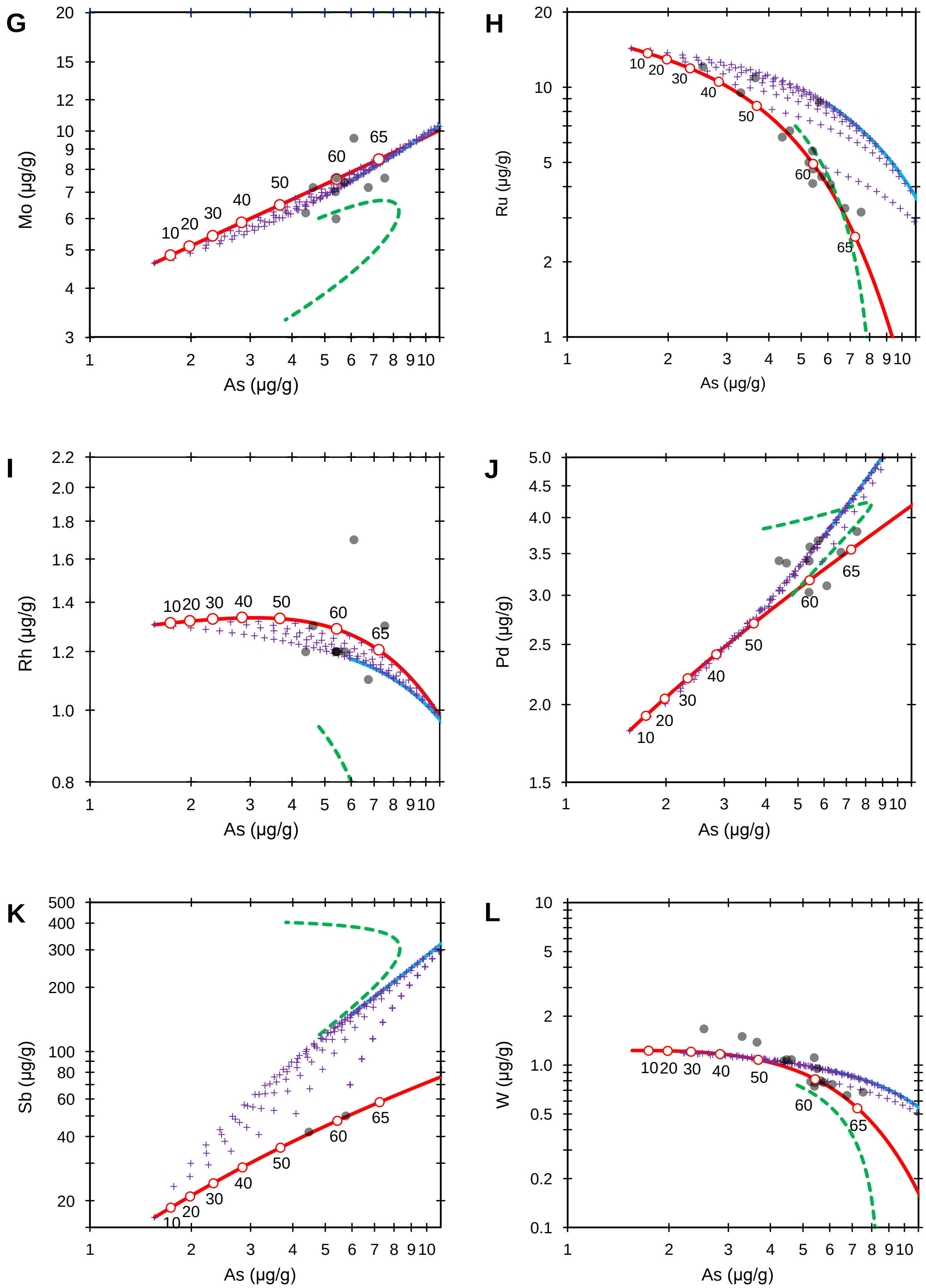
G
Mo (µg/g)
As (µg/g)
H
Ru (µg/g)
As (µg/g)
I
Rh (µg/g)
As (µg/g)
J
Pd (µg/g)
As (µg/g)
K
Sb (µg/g)
As (µg/g)
L
W (µg/g)
As (µg/g)

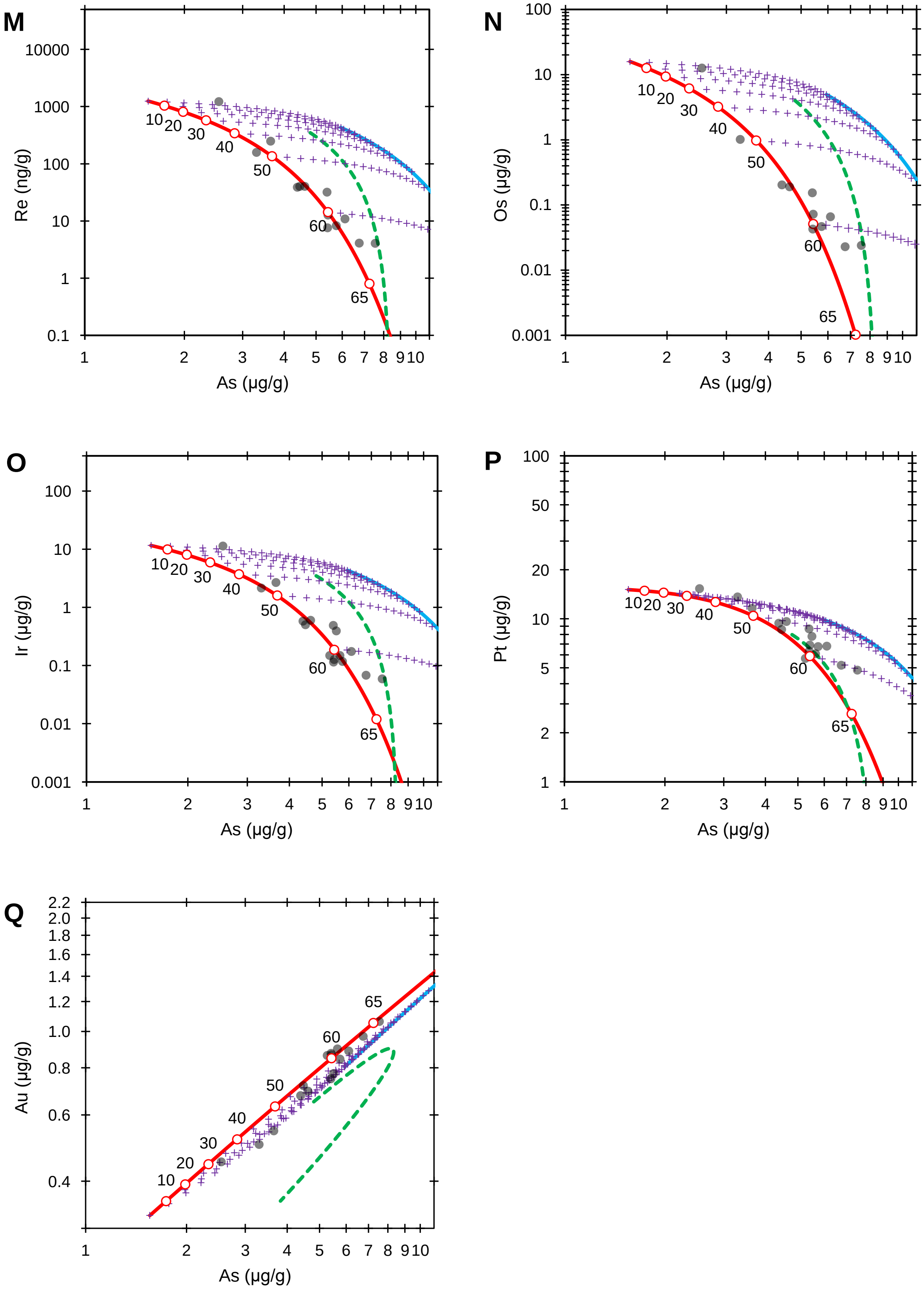

Figure S4. Fractional crystallization modeling of elements-versus-As trends in group IIIE. The figures show results from the optimal bulk 8 wt.% S and 0.50 wt.% P. The black dots are NAA or ICPMS data. The red, blue, and green dashed lines are the solid from simple fractional

crystallization (*SFC solid*), solid from trapped melt (*TM solid*), and liquid (*Liquid*), respectively. The purple crosses are the equilibrium mixing (*Mixing line*) between SFC and trapped-melt solids at an increment of 5%. The labeled circles on the red lines represent the crystallization sequence (*Cryst%*).

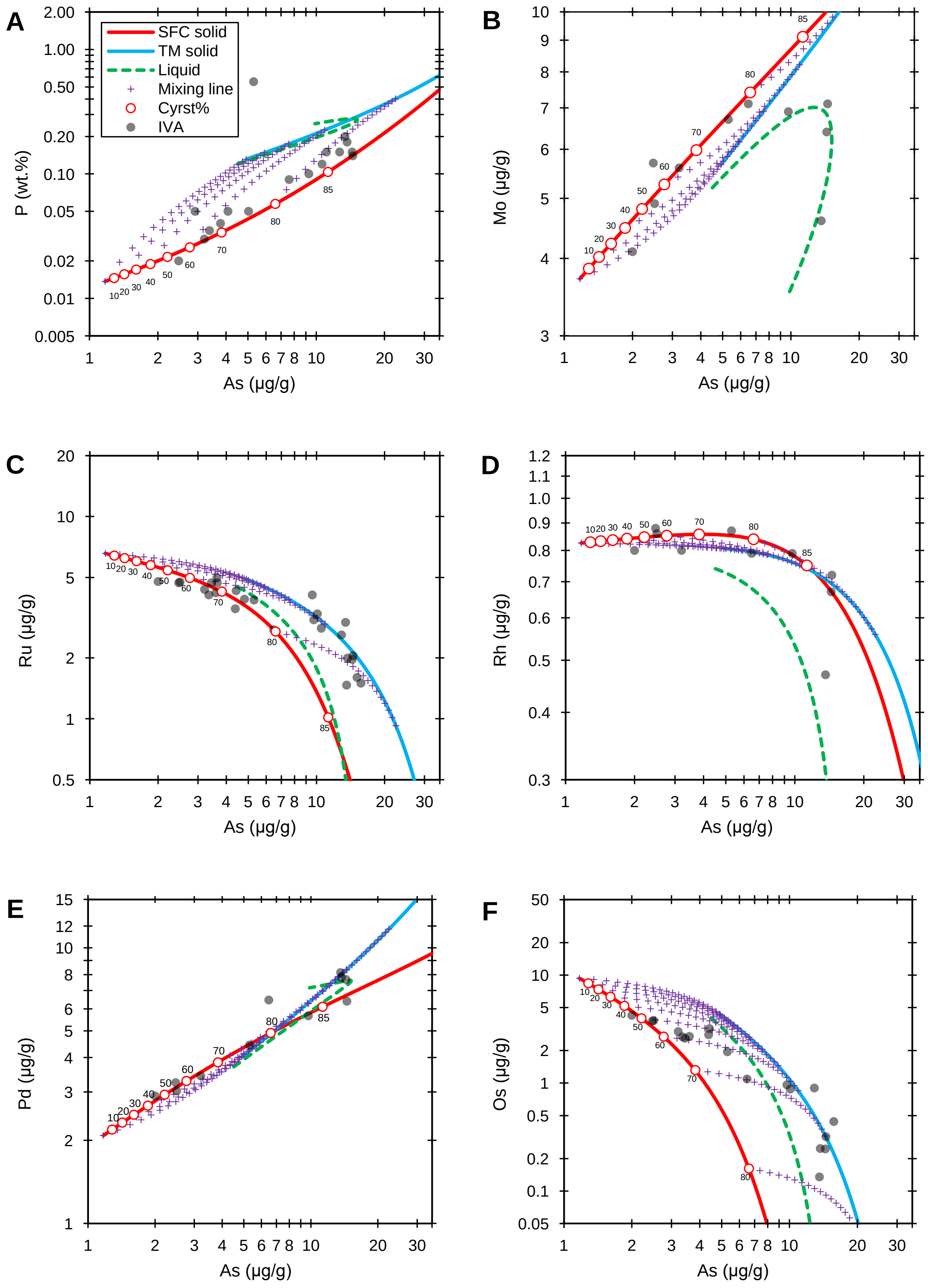
A
SFC solid
TM solid
Liquid
Mixing line
Cyrst%
IVA
P (wt.%)
As (µg/g)
B
Mo (µg/g)
As (µg/g)
C
Ru (µg/g)
As (µg/g)
D
Rh (µg/g)
As (µg/g)
E
Pd (µg/g)
As (µg/g)
F
Os (µg/g)
As (µg/g)

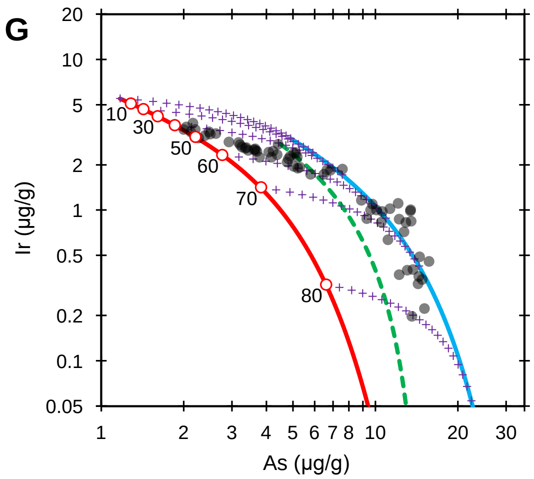

Figure S5. Fractional crystallization modeling of additional elements-versus-As trends in group IVA. The figures show results from the optimal bulk 3 wt.% S and 0.11 wt.% P. The red, blue, and green dashed lines are the solid from simple fractional crystallization (*SFC solid*), solid from trapped melt (*TM solid*), and liquid (*Liquid*), respectively. The purple crosses are the equilibrium mixing (*Mixing line*) between SFC and trapped-melt solids at an increment of 5%. The labeled circles on the red lines represent the crystallization sequence (*Cryst%*).

Estimating CAI abundances in iron-meteorite precursor materials

Previously, the linear relationship between CAI abundance and HSE abundance in carbonaceous chondrites was used to estimate the CAI abundances in CC-iron precursors (11). This method assumes that iron-meteorite precursors are chondrite-like, and CAIs are the main carrier of HSEs in chondrites and iron-meteorite precursors. In this study, we compile all iron groups and estimate the CAI abundance in the precursor of each group.

This study improves the method for estimating CAI abundance. The previous studies normalized siderophile concentrations to Ni (11-13), while we now normalize them to Co (see the reasons in the main text). For carbonaceous chondrites, such a change does not affect the linearity of the relationship between CAI abundance and HSE abundance (linear-regression parameters summarized in Table S1). In Model 1, we used the linear regression prediction band function (95% confidence) in OriginPro. Detailed information for the linear regression method can be found at https://www.originlab.com/doc/Origin-Help/LR-Dialog. In Model 2, we used the Monte Carlo method to do the linear regression (simulation of 10,000 times). We then used these two models to estimate the CAI abundances in iron-meteorite precursor materials (Table S1). The estimated CAI abundances in these two models are almost identical, except that Model 2 has more variable uncertainties.

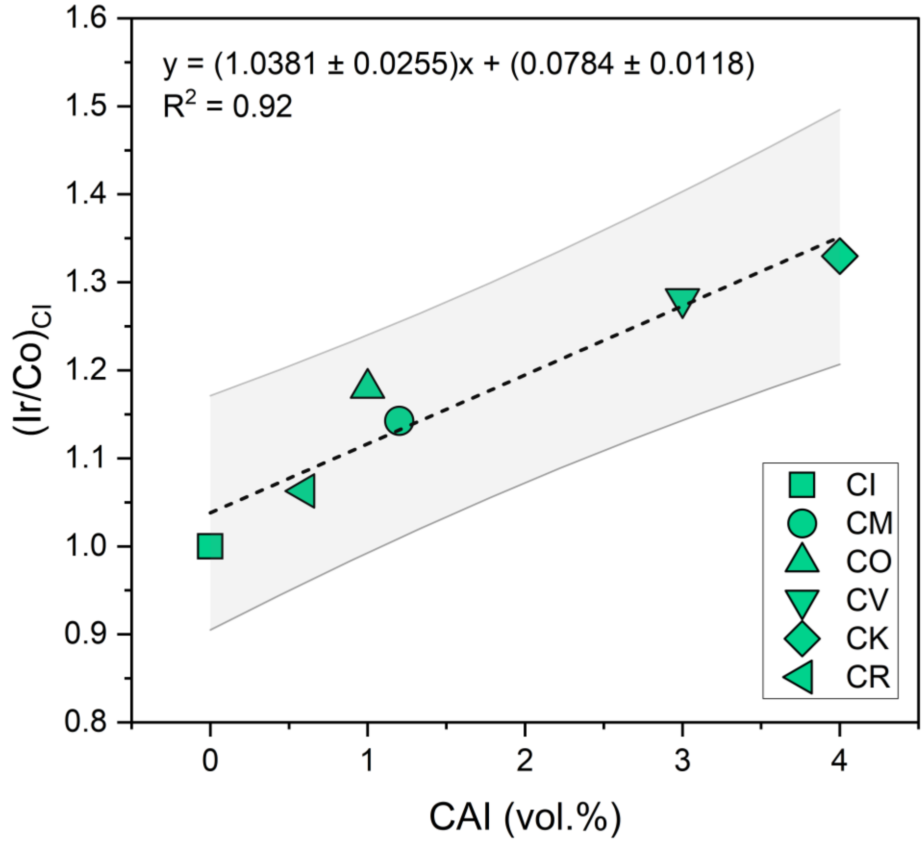

Figure S6. Linear fitting between $(Ir/Co)_{CI}$ values and CAI abundance in carbonaceous chondrites. Cobalt and Ir concentrations (14-16) and CAI abundances (17) of carbonaceous chondrites are from the literature. The linear regression is performed by the Fitted Curve Plot Analysis function in OriginPro. The prediction band predicts that within the 95% confidence level, expected data points are expected to fall within this interval.

Using the new linear relationships between CAI abundance and Ir abundance (Figure S6), we calculated CAI abundances in the iron-meteorite precursors. Iridium is used as a representative element for HSEs because it is one of the most accurately and precisely analyzed elements by INAA. The linear aggression analysis in Fig. S6 was performed by OriginPro, and the estimated CAI abundances of iron-meteorite precursors are shown at 95% confidence intervals (Table S2).

For the CC-iron groups, the CAI abundances derived from this study and the previous study (11) do not show a drastic difference. In the new estimates, the CAI abundance decreases in the IID precursors by ~10 wt.%. The bulk Ni content may be underestimated in IID. For example, the formation of some Ni-rich phases was not considered by the fractional-crystallization models. Despite the difference, the modification to the estimates does not change our overall conclusions on the CAI distribution in the disk, the formation locations of iron-meteorite parent bodies, and the origin of HSEs in these two groups.

Table S1. Comparison of different models of CAI abundance estimation.

Group/ Groupilet*		Based on $(Ir/Ni)_{CI}$†		Based on $(Ir/Co)_{CI}$ (Model 1)		Based on $(Ir/Co)_{CI}$ (Model 2)	
		CAI (vol.%)	CAI (wt.%)‡	CAI ± 2σ (vol.%)	CAI ± 2σ (wt.%)‡	CAI ± 2σ (vol.%)	CAI ± 2σ (wt.%)‡
NC	IC	N.A.	N.A.	0	0	0	0
	IIAB	N.A.	N.A.	6 ± 6	8 ± 8	6 ± 7	8 ± 9
	IIIAB	N.A.	N.A.	0	0	0	0
	IIIE	N.A.	N.A.	0	0	0	0
	IVA	N.A.	N.A.	0	0	0	0
CC	IIC	0	0	0	0	0	0
	IID	21	27	12 ± 6	16 ± 8	11 ± 8	15 ± 11
	IIF	8	11	7 ± 6	10 ± 8	6 ± 7	9 ± 9
	IIIF	N.A.	N.A.	13 ± 6	17 ± 8	12 ± 8	17 ± 12
	IVB	20	26	21 ± 6	27 ± 8	20 ± 10	26 ± 14
	SBT	0	0	0	0	0	0

*Some groups show negative values of the CAI estimates. Negative estimates are listed as 0 vol.%.
†Data from Ref (11); N/A = not applicable.
‡The weight percent of CAIs is calculated based on volume percent assuming the precursor material of iron meteorites is composed of CAI and CI chondrite-like host matrix, which have densities of 3.1 g/cm^3 (18) and 2.2 g/cm^3 (19), respectively.

Empirical T_{50} estimate for Au

In Figure S7, the normalized Au abundances form a hump compared to adjacent elements. The normalized Au abundances of all groups are also much less scattered compared to adjacent Ga and Ge. Our model-derived data show that Au may have a T_{50} between those of Pd (1330 K) and As (1235 K), and this empirical estimate is consistent with the T_{50} of 1284 K for Au derived from elemental abundances of ordinary chondrites (20). In Figures 2, we used this empirical T_{50} estimate for Au.

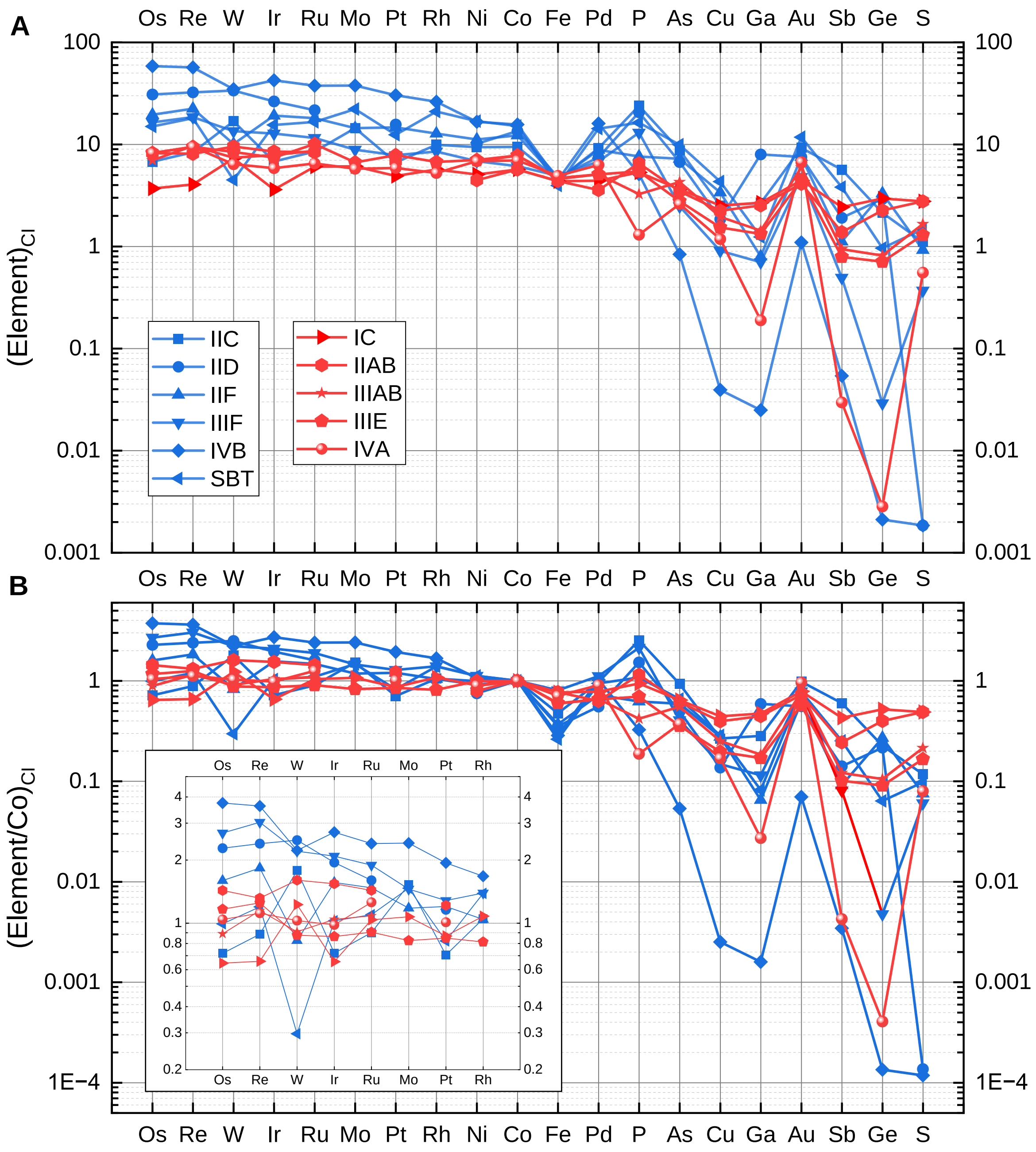

Figure S7. Bulk siderophile abundances of asteroidal cores. **A**: Siderophile concentrations normalized to CI chondrites (21). **B**: Siderophile concentrations normalized to Co and CI chondrites. The small panel in Figure S7B shows the magnified plot of Os, Re, W, Ir, Ru, Mo, Pt and Rh. Data of groups IIC, IID, IIF, IVB, and the SBT from Ref (11), IIIAB from Ref (7) with addition of Ru and Pd from this work, IIIF from Ref (22), and IVA from Ref (23) with addition of Os, Ru, Mo, Pd, and Rh from this work. Siderophile elements are ordered by decreasing T_{50} (24) from left to right.

Table S2. Compositions reported in this study for groups IC, IIAB, IIIAB, IVA and IIIE. Concentrations in µg/g unless noted.

Meteorite	Cr	Co (mg/g)	Ni (mg/g)	Cu	Ga	Ge	As	Mo	Ru	Rh	Pd	Sb (ng/g)	W	Re (ng/g)	Os	Ir	Pt	Au	P* (%)
**IC**																			
Arispe	79	4.67	62.9	118	51.9	246	7.25	*7.4*	***13.22***	*1.9*	***2.897***	99	2.30	***773.8***	***6.737***	10.07	***18.75***	0.811	0.30
Nova 057	36	4.65	67.0	120	50.7	211	7.55		13.6				1.98	961	7.5	9.87	18.6	0.878	
Union County	136	4.59	63.1	167	55.7	216	4.78	*7.7*	***13.19***	*2.1*	***1.705***		2.14	***145.6***	***1.022***	2.37	***18.71***	0.662	0.20
Park City	66	4.56	58.9	127	57.0	178	5.15						2.24	151		2.32	21.3	0.661	
Murnpeowie	244	4.46	65.2	167	41.9	85.4	4.61						1.65	170		2.19	10.9	0.622	0.20
Mount Dooling	124	4.59	62.6	160	54.8	234	4.77	*11.3*	***11.91***	*1.9*	***2.118***		2.00	***71.52***	***0.368***	1.25	***17.38***	0.657	0.27
Bendego	55	4.64	65.2	164	55.2	241	5.84	*6.3*	***10.78***	*2.0*	***2.269***		1.51	***15.47***	***0.1397***	0.212	***15.34***	0.754	0.22
Northwest Africa 2743	53	4.76	67.5	137	52.6	195	8.28	*9.0*	***4.70***	*1.6*	***2.966***		0.88	***9.8335***	***0.07118***	0.127	***6.16***	0.971	
Etosha	71	4.78	67.1	146	50.1	217	8.09	*7.1*	***3.643***	*1.4*	***2.583***		0.75	***9.980***	***0.1248***	0.118	***4.268***	0.993	0.33
Chihuahua City	59	4.73	68.1	149	52.4	212	7.70	*8.7*	***3.773***	*1.6*	***3.185***		0.82	***9.774***	***0.08927***	0.108	***5.174***	0.981	0.39
St. Francois County	44	4.80	64.8	115	50.2	237	8.58	*6.3*	***8.195***	*1.5*	***3.140***		1.36	***5.817***	***0.03964***	0.105	***11.32***	0.976	0.34
Santa Rosa	93	4.72	65.2	137	50.2	222	6.91	*7.9*	***4.074***	*1.4*	***3.905***		0.91	***4.671***	***0.03568***	0.066	***6.03***	0.925	0.36
Elton	18	4.97	70.5	136	48.9	165	11.7		3.4			166	0.51			0.060	3.8	1.391	
**IC-an**																			
Nocoleche	130	4.48	63.3	169	48.8	148	4.18	*7.1*	***11.22***	*1.6*	***2.033***		1.68	***657.4***	***7.329***	7.64	16.3	0.573	
Winburg	50	4.77	70.3	110	49.3	180	9.72	*7.3*	***6.54***	*1.8*	***2.892***	116	1.07	***69.63***	***0.3757***	0.961	8.7	1.069	
**IIAB**																			
Avce	**161**	**4.46**	**54.5**	**148**	**58.8**	**182**	**4.02**						**4.00**	**7309**		**59.5**	**39.4**	**0.514**	0.20
Uasara	72	4.45	55.5	141	59.5	178	3.76		29.7				3.73	5610	***63.3***	48.2	37.5	0.479	
Scottsville	**86**	**4.48**	**53.7**	**141**	**60.4**	**172**	**3.79**						**3.91**	**5210**		**47.9**	**35.7**	**0.492**	0.21
Negrillos	**92**	**4.49**	**52.5**	**136**	**57.5**	**179**	**3.55**		***27.60***		*1.607*		**3.72**	**4894**	***63.581***	**47.4**	**33.1**	**0.477**	0.22
Patos de Minas (hexahedrite)		**4.47**	**53.2**	**142**	**58.4**	**170**	**3.62**					**36**	**3.62**	**4928**		**46.3**	**36.5**	**0.486**	0.20
Sierra Gorda	**144**	**4.49**	**55.4**	**156**	**60.3**	**169**	**3.64**						**3.86**	**4890**		**43.9**	**35.9**	**0.488**	0.23
Bennett County	**92**	4.43	**54.0**	**131**	**56.5**	**179**	**3.53**		***27.8***		*1.610*		**3.62**	**4540**	***56.349***	**43.6**	**31.8**	**0.474**	0.23
Guadalupe y Calvo	**68**	**4.47**	**54.0**	**136**	**60.2**	**182**	**3.91**					**48**	**3.52**	**4520**		**42.6**	**37.2**	**0.534**	0.30
Forsyth County	**63**	**4.44**	**55.4**	**136**	**60.4**	**176**	**3.90**		***26.46***		*1.634*		**3.69**	**4044**	***44.079***	**40.4**	**35.1**	**0.510**	0.21
Smithonia	**69**	**4.52**	**56.0**	**122**	**59.3**	**187**	**3.94**						**3.65**	**3802**		**38.3**	**35.1**	**0.537**	0.20
Murphy	**64**	**4.48**	**53.3**	**143**	**60.4**	**186**	**3.93**						**3.81**	**3880**		**37.8**	**34.2**	**0.506**	0.34
Crow Peak	**128**	**4.46**	**57.2**	**138**	**57.8**	**167**	**3.72**		**27.4**				**3.40**	**3365**		**36.5**	**34.5**	**0.515**	
Edmonton (Canada)	**89**	**4.51**	**53.1**	**149**	**59.5**	**172**	**3.77**						**3.61**	**2928**		**31.5**	**35.6**	**0.513**	0.20
Pirapora	**99**	**4.51**	**54.1**	**137**	**58.9**	**189**	**3.83**						**3.51**	**3070**		**31.0**	**34.4**	**0.523**	0.20
Pirapora (Angra dos Reis)	107	4.49	53.4	134	56.9	188	3.89						3.50	2790		30.8	33.2	0.524	
Arraias	124	4.50	54.2	133	57.4	<200	3.79		24.9			<150	3.46	2940	25.9	30.8	32.0	0.512	
Bruno	**157**	**4.51**	**53.6**	**124**	**58.0**	**185**	**3.86**						**3.48**	**2835**		**30.2**	**32.1**	**0.509**	0.20
Allan Hills A78100	**77**	**4.48**	**54.9**	**141**	**58.3**	**188**	**3.87**					**43**	**3.50**	**2829**		**30.0**		**0.522**	0.20
San Francisco del Mezquital	**116**	**4.49**	**55.1**	**138**	**60.2**	**183**	**4.11**						**3.47**	**2770**		**28.5**	**31.0**	**0.522**	0.20
Boguslavka	**70**	**4.48**	**54.6**	**140**	**59.2**	**180**	**4.01**						**3.33**	**2405**		**25.9**	**31.5**	**0.523**	0.30
Northwest Africa 2678	**106**	**4.47**	**54.5**	**134**	**58.0**	**163**	**3.83**		**27**				**3.17**	**2370**		**25.7**	**30.6**	**0.527**	0.25
Cosmo Newberry	**83**	**4.61**	**54.3**	**116**	**59.3**	**180**	**4.08**					**57**	**3.55**	**2100**		**23.2**	**30.6**	**0.538**	0.20
Larkman Nunatak 12204	74	4.50	55.2	140	57.8	158	3.80		23.7			148	3.27	1394	***17.4***	22.8	32.1	0.513	
Cincinnati	**74**	**4.53**	**54.5**	**145**	**58.2**	**178**	5.20		***23.70***		*1.706*		**3.43**	**1720**	***13.328***	**20.3**	**30.0**	**0.540**	0.20
Holland's Store	**55**	**4.51**	**54.5**	**135**	**59.9**	**182**	**4.01**						**3.46**	**1545**		**18.4**	**29.2**	**0.529**	
Cruz Alta	74	4.52	56.1	140	60.5	216	4.38		22.4				3.2	1510	***11.30***	16.4	27.1	0.555	
Coahuila	**214**	**4.54**	**55.0**	**141**	**58.5**	**180**	**4.20**		***22.44***		*1.746*		**3.15**	**1290**	***9.8385***	**16.1**	**28.30**	**0.546**	0.28
Yarroweyah	**216**	**4.51**	**56.3**	**148**	**60.8**	**171**	**4.20**						**3.29**	**1510**		**16.0**	**29.8**	**0.552**	
Anyujskij	**56**	**4.47**	**55.1**	**138**	**59.5**	**172**	**4.09**					**41**	**3.18**	**1410**		**14.4**	**26.0**	**0.546**	
Mayodan	80	4.54	54.4	130	58.0	180	4.08						3.06	1113		14.2	24.8	0.541	
Muenatauray	**132**	**4.52**	**53.9**	**130**	**57.5**	**183**	**4.08**						**3.06**	**1046**		**12.9**	**27.6**	**0.553**	
Keen Mountain	**101**	**4.54**	**54.9**	**139**	**59.3**	**182**	**4.32**						**3.13**	**1004**		**12.1**	**25.8**	**0.554**	
Indian Valley	**94**	**4.49**	**54.6**	**144**	**58.5**	**174**	**4.32**						**3.01**	**954**		**11.6**	**28.6**	**0.560**	0.27
Northwest Africa 3202	138	4.51	56.1	131	58.9		4.14		24.3				2.82	853		11.4	28.4	0.570	
Northwest Africa 11446	84	4.50	56.1	128	58.0	153	4.16		21.4			<100	3.12	943	5.8	11.4	26.6	0.548	
Okano	**233**	**4.55**	**56.0**	**138**	**59.9**	**180**	**4.34**						**3.03**	**859**		**11.4**	**25.5**	**0.587**	
Braunau	**73**	**4.52**	**55.0**	**141**	**59.7**	**183**	**4.36**		***21.20***		*1.778*		**3.16**	**877**	***5.527***	**11.3**	**30.4**	**0.572**	0.24
Carver	**77**	**4.46**	**56.8**	**138**	**59.4**	**184**	**4.08**						**3.06**	**863**		**11.3**		**0.555**	
Siratik	**61**	**4.45**	**56.6**	**136**	**57.2**	**188**	**4.11**					**51**	**3.09**	**958**		**11.2**		**0.550**	
Okahandja	**86**	**4.53**	**55.9**	**144**	**57.8**	**193**	**4.03**						**3.08**	**845**		**10.9**	**29.9**	**0.537**	
Squaw Creek	**65**	4.50	**54.9**	**126**	**57.8**	**182**	**4.21**					**47**	**3.21**	**952**		**10.6**		**0.544**	0.28
Miller Range 07666	54	4.56	54.8	132	58.0	187	4.23		21.4			<150	2.77	768	5.4	10.3	24.8	0.559	
Cedartown	**96**	**4.56**	**54.5**	**132**	**59.5**	**181**	**4.35**						**2.88**	**830**		**10.2**	**25.7**	**0.559**	0.30
Nova 057	36	4.65	67.0	120	50.7	211	7.55		13.6			<100	1.98	961	7.5	9.87	18.6	0.878	
Calico Rock	**77**	**4.51**	**55.6**	**135**	**59.0**	**186**	**4.32**						**3.01**	**741**		**9.27**	**26.4**	**0.578**	0.28
Pima County	**71**	**4.56**	**56.5**	**149**	**60.6**	**182**	5.14						**2.96**	**663**		**8.80**	**26.5**	**0.586**	0.25
Richland	**36**	**4.56**	**55.3**	**125**	**59.9**	**181**	**4.65**						**2.75**	**660**		**8.52**	**26.6**	**0.586**	0.24
Fredricksburg	**91**	**4.58**	**55.2**	**134**	**58.4**		**4.38**						**2.83**	**666**		**8.44**	**25.2**	**0.575**	
Chico Mountains	**58**	**4.52**	**54.1**	**133**	**58.4**	**176**	**4.29**						**2.78**	**622**		**8.41**	**24.4**	**0.558**	0.33
Wathena	**58**	**4.53**	**54.6**	**136**	**59.0**	**184**	**4.40**						**2.85**	**599**		**8.02**	**24.7**	**0.586**	0.27
Northwest Africa 8302	98	4.55	57.0	133	59.7	234	4.58		19.6			115	2.78	523	3.1	7.59	24.85	0.590	
Gressk	**41**	**4.58**	**55.7**	**137**	**60.4**	**177**	**4.58**		***18.47***		*1.803*		**2.76**	**561**	***3.2374***	**7.48**	**24.3**	**0.598**	0.28
Locust Grove	**69**	**4.54**	**56.1**	**134**	**59.7**	**180**	**4.64**						**2.74**	**566**		**7.46**	**22.8**	**0.592**	0.25
Santo Antônio do Descoberto	500	4.56	57.6	138	60.1	144	4.61		18				2.72	456	***2.3***	6.03	24.0	0.594	
El Mirage	**76**	**4.58**	**55.4**	**137**	**58.6**	**185**	**4.52**						**2.78**	**405**		**5.93**	**23.9**	**0.593**	
Hex River Mountains	**123**	**4.57**	**55.8**	**131**	**59.1**	**181**	**4.65**						**2.48**	**279**		**4.13**	**22.8**	**0.609**	0.25
Bingera	37	4.57	56.1	135	59.0	173	4.75					<150	2.48	222		3.44	22.3	0.623	
Kopjes Vlei	**78**	**4.56**	**55.9**	**141**	**59.8**	**182**	**4.96**						**2.46**	**225**		**3.43**	**21.2**	**0.630**	0.30
North Chile	46	4.56	55.9	130	58.9	176	4.74		***17.7***		*1.854*	78	2.60	223	***1.0045***	3.36	**21.8**	0.620	0.30
Walker County	**152**	**4.60**	**55.3**	**135**	**58.6**	**189**	**4.86**						**2.50**	**233**		**3.27**	**22.0**	**0.623**	0.28
Uwet	**73**	**4.59**	**56.6**	**136**	**59.4**	**182**	**4.94**						**2.39**	**192**		**2.77**	**18.7**	**0.629**	0.25
Lombard	**46**	**4.58**	**55.0**	**129**	**59.0**	**177**	**5.09**						**2.32**	**196**		**2.68**	**20.7**	**0.645**	0.28
Yamato 75105	46	4.87	59.1	132	58.2	170	4.30						2.11	290		2.50		0.700	
Northwest Africa 11637	154	4.64	62.6	184	56.4	194	4.89		12.6			<70	2.20	188	1.0	2.35	16.1	0.637	
Park City	**66**	**4.56**	**58.9**	**127**	**57.0**	**178**	**5.15**						**2.24**	**151**		**2.31**	**21.3**	**0.661**	
Elqui	**590**	**4.57**	**59.8**	**136**	**59.3**	**167**	**5.23**					**65**	**2.16**	**140**		**1.94**	**17.3**	**0.662**	

Mejillones	**178**	**4.62**	**54.9**	**130**	**59.7**	**177**	**5.16**						**2.22**	**130**		**1.92**	**19.2**	**0.663**	0.33
Chesterville	**201**	**4.61**	**56.3**	**136**	**59.4**	**178**	**5.22**						**2.26**	**130**		**1.77**	**19.8**	**0.654**	0.34
Old Woman	**47**	**4.66**	**55.4**	**125**	**57.0**	**184**	**5.48**						**2.05**	**44.7**		**0.830**	**15.2**	**0.689**	
Navajo	**37**	**4.69**	**54.9**	**126**	**58.3**	**181**	**5.96**						**1.89**	**28.2**		**0.513**	**14.6**	**0.719**	0.30
Mount Joy	**52**	**4.66**	**57.8**	**133**	**60.5**	**182**	**6.22**						**1.61**	**22.8**		**0.423**	**12.9**	**0.779**	0.28
Jerslev	**38**	**4.79**	**55.4**	**124**	**57.7**	**168**	**6.99**					**59**	**1.26**			**0.234**	**9.9**	**0.830**	0.35
Sandia Mountains	**44**	**4.75**	**57.9**	**130**	**57.7**	**170**	**7.62**						**1.24**	**8.44**		**0.136**	**8.8**	**0.879**	0.40
Bilibino	**36**	**4.71**	**59.9**	**127**	**59.7**	**176**	**7.43**					**66**	**1.39**			**0.121**	**9.3**	**0.86**	
El Burro	**38**	**4.73**	**58.8**	**121**	**54.8**	**168**	**7.27**						**1.03**			**0.077**	**8.4**	**0.887**	0.32
Nenntmannsdorf	**23**	**4.80**	**59.9**	**130**	**56.7**	**172**	**7.74**					**87**	**1.08**			**0.077**	**8.0**	**0.903**	0.30
Smithsonian Iron	**38**	**4.85**	**55.5**	**109**	**54.0**	**165**	**7.38**						**1.10**	**3.89**		**0.065**	**7.7**	**0.873**	0.25
Iredell	**28**	**4.78**	**58.9**	**123**	**57.1**	**163**	**8.36**						**0.92**			**0.045**	**7.1**	**0.970**	0.20
Veevers	**26**	**4.81**	**57.2**	**117**	**56.0**	**160**	**8.74**						**0.89**			**0.029**	**4.9**	**0.998**	
Sikhote-Alin	**31**	**4.79**	**60.3**	**117**	**53.7**	**158**	**9.40**						**0.74**			**0.024**	**5.5**	**1.049**	
Xifu	18	4.71	61.4	151	54.4	140	8.88		4.1				0.75			0.024	4.2	1.043	
Ainsworth	**32**	**4.84**	**61.4**	**115**	**54.0**	**142**	**9.25**						**0.64**	1.51		**0.022**	**4.1**	**1.068**	0.70
Dutch Flat	**38**	**4.89**	**57.8**	**112**	**55.5**	**132**	**9.04**						**0.82**			**0.021**	**5.2**	**1.030**	0.50
Foum Zguid	**22**	**4.97**	**58.1**	**113**	**55.5**	**153**	**9.91**						**0.72**			**0.021**	**5.3**	**1.078**	
Northeast Africa 007	31	4.80	63.4	122	53.6	151	8.94		5.40			<150	0.78	<19	<0.2	0.020	4.7	1.058	
Derrick Peak A78009	**28**	**4.76**	**63.6**	**113**	**52.9**	**135**	**9.31**					**94**	**0.56**			**0.018**	**4.1**	**1.084**	
Northwest Africa 3201	**15**	**5.11**	**52.1**	**118**	**51.6**	**130**	**11.1**		**2.5**				**0.54**			**0.017**	**3.7**	**1.089**	
Agoudal	22	4.75	63.7	128	58.6	164	9.58		3.50			91	0.77		0.23	0.015	5.5	1.036	
Lake Murray	**26**	**5.00**	**60.3**	**106**	**52.7**	**141**	**10.2**						**0.56**			**0.015**	**2.7**	**1.087**	0.50
Summit	**17**	**4.84**	**65.5**	**116**	**50.4**	**115**	**11.4**						**0.42**			**0.014**	**1.9**	**1.267**	0.50
Santa Luzia	**15**	**5.05**	**60.4**	**107**	**46.2**	**110**	**11.7**					**100**	**0.33**	**0.714**		**0.013**	**2.9**	**1.290**	0.90
São Julião de Moreira	**16**	**5.19**	**57.8**	**94**	**45.8**	**106**	**12.5**					**79**	**0.40**	**0.765**		**0.012**	**2.3**	**1.350**	0.90
Silver Bell	**15**	**5.26**	**58.9**	**98**	**45.7**	**111**	**12.3**						**0.36**			**0.012**	**1.9**	**1.252**	0.80
**IIAB-an**																			
Elephant Moraine 83245	**22**	**4.86**	**60.4**	**119**	**55.8**	**157**	**9.71**					**86**	**0.74**	**<100**		**0.026**		**1.065**	
**IIIAB**																			
Costilla Peak									***12.740***		***3.349***								
Henbury									***13.180***		***2.127***								
Charcas									***8.411***		***2.619***								
Tamarugal									***4.854***		***3.336***								
Maldyak									***4.602***		***4.325***								
**IVA†**																			
Huangling	388	3.75	77.5	146	1.84	0.179	2.27					38	0.85	386		3.01		0.645	
Christina	267	3.90	78.1	156	1.77		3.63		5.8				0.54	286	2.6	2.52	6.1	0.888	
South African Railways	160	3.89	79.6	162	1.98		3.60		4.2				0.51	287	2.7	2.52	6.2	0.880	
Nova 019	260	3.89	75.3	142	2.16		3.64		3.8				0.59	302	2.7	2.49	5.0	0.917	
Serrania de Varas	124	3.91	81.1	125	2.15	0.131	4.81		3.9				0.63	242		2.19	6.0	1.169	
Parkajoki	44	4.14	97.4	671	2.41		11.4		3.3				1.96	142	1.6	1.21	4.6	2.211	
Novorybinskoe	272	4.15	93.1	108	2.35	0.202	13.5		3.0				0.41	102		1.01	4.4	2.373	
Miller Range 03356	20	4.07	88.2	101	2.17		9.59		4.1				0.39	92		1.00	4.3	1.983	
Miller Range 07119	69	4.06	89.9	110	2.25		10.1		3.3				0.43	103	0.88	1.00	4.7	1.997	
Larkman Nunatak 06877	9	4.07	88.3	122	2.24		10.5		2.8				0.40	86		0.830	4.2	1.994	
**IVA-an**																			
Longchang	385	4.27	84.0	154	2.03	0.400	4.68					4763	1.21	517		4.65		0.577	
Zaragoza	199	4.14	77.2	153	2.12		3.85		4.5				0.59	298	3.03	2.50	5.5	0.973	
**IIIE**																			
Piedade do Bagre	276	4.67	75.6	147	15.4	25.7	2.54		12.1				1.67	1217	12.6	11.3	15.3	0.450	
Cachiyuyal	191	4.76	79.5	137	16.4	30.3	3.65		10.9				1.38	248		2.660	11.6	0.544	0.15
Murfreesboro	154	4.73	78.3	148	17.1	30.0	3.30		9.5				1.50	158	1.0	2.14	13.6	0.501	
Coopertown	140	4.84	83.2	137	16.7	34.9	4.62	*7.2*	***6.70***	*1.3*	***3.38***		1.08	***40.24***	***0.19***	0.599	***9.60***	0.694	0.19
Kokstad	81	4.86	81.9	138	17.2	35.9	4.39	*6.2*	***6.31***	*1.2*	***3.41***		1.06	***38.86***	***0.20***	0.578	***9.39***	0.676	0.22
Aliskerovo	82	4.87	83.3	141	17.9	32.8	4.47					42	1.08	40		0.498	8.6	0.718	
Burlington	471	4.85	83.3	142	18.3	36.0	5.40	*7*	***5.56***	*1.2*	***3.03***		1.11	***31.89***	***0.15***	0.487	***8.67***	0.748	0.23
Paneth's Iron	55	4.92	83.6	135	17.4	34.1	5.51						0.95			0.393	7.8	0.773	0.30
Tanokami Mountain	46	5.00	86.1	144	17.8	34.6	6.11	*9.6*	***4.08***	*1.7*	***3.11***		0.76	***10.91***	***0.07***	0.174	***6.79***	0.887	0.17
Northwest Africa 4704	39	4.89	89.0	154	17.5		5.27		5.0				0.79			0.149	5.7	0.863	
Porto Alegre	71	4.96	90.2	146	18.2		5.65		8.7				0.80			0.148	6.1	0.898	
Staunton	71	4.97	84.9	113	17.7	36.6	5.43	*7.6*	***4.71***	*1.2*	***3.59***		0.78	***12.73***	***0.07***	0.127	***6.89***	0.866	0.31
Paloduro	191	4.89	85.8	129	18.3	37.7	5.75	*7.4*	***4.39***	*1.2*	***3.67***	50	0.78	***8.267***	***0.05***	0.117	***6.74***	0.844	0.30
Rhine Villa	54	4.85	88.5	134	18.4	36.3	5.41	*6.0*	***4.12***	*1.2*	***3.41***		0.74	***7.608***	***0.04***	0.115	***6.33***	0.874	0.29
Willow Creek	45	4.92	88.6	127	18.6	36.4	6.75	*7.2*	***3.27***	*1.1*	***3.52***		0.65	***4.095***	***0.02***	0.068	***5.19***	0.970	0.35
Colonia Obrera	35	4.90	88.6	134	18.4	39.7	7.54	*7.6*	***3.17***	*1.3*	***3.8***		0.68	***4.072***	***0.024***	0.059	***4.84***	1.062	0.20
**IIIE-an**																			
Aletai [Ulasitai]	35	5.21	96.9	108	16.7	<55	15.0		2.2			<150	0.28	40	<0.4	0.235	1.9	1.894	
Aletai [Wuxilike]	45	5.20	98.0	105	17.0	<50	15.8		2.6			<150	0.26	16	0.36	0.234	2.6	1.862	
Aletai [Xinjiang (b)]	46	5.15	97.8	109	16.9	42.2	14.4					77	0.29	25		0.228	2.4	1.811	
Aletai [Akebulake]	50	5.17	98.8	110	16.9	<50	15.1		<1.7			<150	0.32	<40	<0.29	0.224	2.0	1.814	

Values in italics, bold text, and bold italics are LA-ICP-MS, NAA, and ID-ICP-MS data, respectively, from the literature. LA-ICP-MS and ID-ICP-MS data are from the literature: IC (25), IIAB and IIIAB (26), IVA (23), and IIIE (27). Some NAA data of IIAB are from Ref (28).

*P concentrations are from modal analysis (10).

†These are new IVA data. The majority of IVA data were published in Ref (23).

Table S3. Replicate NAA data for groups IC, IIAB, IVA and IIIE irons. Concentrations in µg/g unless noted.

Meteorite	Date		Mass (mg)	Cr	Co (mg/g)	Ni (mg/g)	Cu	Ga	Ge	As	Ru	Sb (ng/g)	W	Re (ng/g)	Os	Ir	Pt	Au
	Y	M																
**IC**																		
Arispe	02	10	504	80	4.69	60.9	118	51.6	198	7.19		99	2.22	911		10	19.3	0.798
Arispe	02	10	470	78	4.64	64.9	119	52.2	227	7.31		112	2.37	967		10.09	19.9	0.823
Bendego	77	5	1100	439	4.64	66.2	155	57.7		5.55			1.60	<240		0.250		0.809
Bendego	78	6	469	44	4.65	67.7	146	53.8		5.73	nd	196	1.36	<580		0.204		0.850
Bendego	82	12	695	62	4.64	64.5	155	55.1		5.76			1.66	<89		0.207		0.792
Bendego	99	9	541	56	4.63	64.2	155	54.3	180	5.63		<150	1.44	<40		0.213		0.732
Bendego	03	9	658	57	5.17	64.9	157	55.3	241	5.79		<100	1.54	<30		0.210	10.5	0.773
Bendego	06	5	579	55	4.66	67.1	175	54.5	241	5.99	17.6	<150	1.31	<68		0.220	11.1	0.756
Chihuahua City	88	1	417	61	4.75	69.3	151	53.4	222	7.67		<100	0.84	<30		0.108	4.1	0.977
Chihuahua City	88	4	551	55	4.70	68.6	147	51.0	220	7.72		<100	0.81	<23		0.108	4.6	0.976
Elton	83	12	265	18	4.90	70.0	153	50.3		11.5			0.50			0.063		1.261
Elton	84	12	613	13	4.98	71.1	149	48.8		11.2			0.55	<80		0.057		1.440
Elton	04	12	491	22	4.90	73.9	131	57.1	185	13.0	4.1	168	0.51	<100		0.065	3.9	1.391
Elton	13	2	692	17	5.03	67.8	111	42.1	121	11.5	3.0	165	0.49	<10	<0.21	0.060		1.400
Etosha	88	6	408	72	4.77	67.6	154	50.9	386.0	8.67		145	0.78	<36		0.119	4.9	0.990
Etosha	88	9	590	69	4.78	65.1	138	49.3	195.0	7.51		<100	0.72	<23		0.118	4.5	0.996
Mount Dooling	00	11	611	106	4.61	59.9	159	53.2	211	4.38		102	2.00	78		1.22	15.1	0.642
Mount Dooling	01	5	659	93	4.61	62.7	156	55.0	261	4.73		<80	2.02	50		1.23	15.1	0.643
Mount Dooling	12	6	444	135	4.58	64.0	162	56.4	243	4.73	13.8	<60	2.03	64	0.56	1.25	14.9	0.655
Mount Dooling	12	6	645	133	4.59	62.7	152	55.7	226	4.86	12.8	<60	1.89	83	0.43	1.27	15.3	0.677
Mount Dooling	12	10	622	138	4.54	63.7	183	56.7	363	4.96	12.6	<120	2.06	88	0.20	1.28	17.7	0.666
Murnpeowie	88	6	585	243	4.46	67.2	171	42.4	90	4.73		<100	1.73	164		2.17	11.4	0.622
Murnpeowie	88	9	668	244	4.45	64.3	163	41.6	99	4.49		<100	1.51	176		2.20	10.4	0.622
Nova 057	19	10	485	36	4.65	67.0	120	50.7	211	7.55	13.6	<100	1.98	961	7.5	9.87	18.6	0.878
Northwest Africa 2743	05	4	661	52	4.75	68.2	142	52.7	217	8.05		88	0.92	<32		0.134	4.9	0.996
Northwest Africa 2743	05	8	641	54	4.76	66.8	132	52.5	170	8.50		<150	0.83	20		0.119	5.8	0.945
Park City	99	4	504	71	4.54	59.6	124	55.1	177	5.02		<200	2.19	161		2.32	20.8	0.635
Park City	99	3	555	60	4.58	58.2	130	58.9	180	5.27		<100	2.29	140		2.31	21.8	0.687
Park City	12	2		144	4.58	63.4	159	55.0	297	4.77	14.2	88	2.03	150	1.07	2.37	18.9	0.666
Santa Rosa	88	1	533	94	4.74	63.9	133	50.2	218	6.75		<100	1.00	<30		0.064	5.1	0.907
Santa Rosa	88	4	383	92	4.70	64.1	141	49.8	222	7.07		<100	0.83	<34		0.067	3.6	0.915
St. Francois County	03	2	483	48	4.79	66.3	120	52.5	204	8.64		<90	1.37	<20		0.100	9.0	0.985
St. Francois County	03	4	463	41	4.79	62.7	114	51.9	210	8.83		<100	1.34	<42		0.109	9.0	0.972
St. Francois County	12	2	499	44	4.82	62.3	111	48.0	296	8.14	7.1	163	1.38	<17	<0.17	0.105	9.3	0.972
Union County	01	5	505	142	4.83	67.4	181	58.2	269	4.86		<100	2.24	204		2.54	19.1	0.771
Union County	01	12	595	107	4.62	59.6	159	56.3	<230	4.45		<150	2.26	157		2.25	16.8	0.631
Union County	12	2		144	4.58	63.4	159	55.0	297	4.77	14.2	88	2.03	150	1.07	2.37	18.9	0.666
Union County	12	10	572	151	4.57	63.1	169	54.3	220	4.89	13.2	<120	2.02	184	0.90	2.30	14.8	0.614
Zaffra	77	1	750	<100	4.81	73.0	147	74.4		14.9			0.66	<174		<0.22		1.648
Zaffra	78	6		21	5.02	69.0	127	65.4		14.9		387	0.78	<49		0.065		1.670
Zaffra	86	4	625	24	4.75	77.2	155	72.9	215	15.0		298	0.48	<32		0.060	4.3	1.661
**IC-an**																		
Nocoleche	88	1	502	120	4.50	62.9	167	47.9	132	4.06		<100	1.67	745		7.56	15.6	0.558
Nocoleche	88	4	428	139	4.46	63.4	171	49.3	162	4.30		<100	1.70	808		7.73	13.2	0.582
Winburg	77	9	900	42	4.85	66.9	118	48.1		8.45			1.15	119		0.874		1.068
Winburg	78	7	738	51	4.82	72.7	82	48.9		12.0			1.12	148		1.030		1.123
Winburg	92	4	492	56	4.69	71.1	113	49.6		9.81			1.02	<90		0.956	8.2	1.083
Winburg	93	2	612	46	4.78	70.3	118	48.5		9.11			1.05	68		0.976	5.8	1.028
**IIAB**																		
Agoudal [Imilchil]	13	4	588	22	4.75	63.7	128	58.6	164	9.58	3.50	91	0.77	<24	0.23	0.015	5.5	1.036
Agoudal [Imilchil]	13	7	614	20	4.82	59.5	110	53.8	179	9.15	3.40	<150	0.70	<50	<0.34	0.021	4.1	1.059
Arraias	18	2	563	179	4.52	53.6	130	57.5	254	3.87	23.9	100	3.51	2970	25.8	30.8	34.4	0.510
Arraias	18	6	508	97	4.49	54.8	136	57.4	173	3.71	26.0	<180	3.42	2905	25.9	30.9	29.6	0.513
Bingera	00	9	502	73	4.57	56.3	135	59.2	158	4.75		<150	2.48	222		3.35	22.3	0.623
Cruz Alta	12	6	454	65	4.51	57.7	131	59.1	211	4.30	22.9	105	3.13	1420	11.4	16.2	25.4	0.546
Cruz Alta	12	10	452	83	4.52	54.4	148	61.8	220	4.46	21.9	<110	3.27	1595	11.1	16.6	28.8	0.564
Larkman Nunatak 12204	16	1	501	75	4.50	51.7	139	56.6	147	3.7	23.5	162	3.17	1968	17.4	22.4	31.5	0.505
Larkman Nunatak 12204	16	4	507	72	4.50	54.8	141	58.9	169	3.8	23.9	135	3.37	1900	17.4	22.9	32.7	0.521
Mayodan	00	6	522	79	4.52	53.7	131	57.1	174	4.02		<150	3.08	1161		14.2	23.8	0.545
Mayodan	00	8	587	81	4.55	54.2	128	57.6	178	4.13		<150	3.03	1065		14.1	25.8	0.537
Miller Range 07666	09	6	409	55	4.54	54.2	137	57.7	183	4.27	19.5	<150	2.78	781		10.5	25.7	0.568
Miller Range 07666	10	1	602	52	4.57	55.3	126	58.2	191	4.18	23.2	<150	2.75	755	5.41	10.0	23.9	0.550
North Chile [San Martin]	83	7	330	48	4.53	56.3	130	59.6		4.96			2.64	224		3.70		0.695
North Chile [San Martin]	83	7	432	48	4.54	55.5	130	59.5		4.62			2.54	234		3.60		0.622
North Chile [San Martin]	84	3	574	49	4.62	57.0	130	59.0		4.70			2.56	240		3.40		0.598
North Chile [San Martin]	84	5	453	58	4.52	56.4	133	62.2		4.84			3.01	249		3.87		0.610
North Chile [San Martin]	84	12	387	50	4.56	54.9	137	57.7		6.05			2.57	237		3.41		0.618
North Chile [San	85	6	455	58	4.50	56.7	134	58.9		5.23			2.76	242		3.32		0.622

Martin]																		
North Chile [San Martin]	85	8	535	62	4.42	56.1	132	58.3		4.74			2.56	239		3.36		0.613
North Chile [San Martin]	85	9	481	31	7.15	57.1	134	58.8		4.81			2.56	224		3.39		0.609
Northeast Africa 007	19	2	437	30	4.81	60.6	123	54.0	144	8.96	5.50	<150	0.68	<39	<0.17	0.020	3.9	1.042
Northeast Africa 007	19	6	485	29	4.76	66.5	124	53.7	153	8.99	5.30	<150	0.90	<19	<0.2	0.019	5.4	1.055
Northwest Africa 11446	16	4	619	71	4.53	56.6	130	58.1	113	4.21	22.0	<150	3.11	960	5.9	11.5	26.2	0.548
Northwest Africa 11446	16	10	517	96	4.46	55.6	126	58.0	153	4.11	20.7	<100	3.14	926	5.8	11.4	27.1	0.549
Northwest Africa 11637	19	2	554	148	4.61	63.7	170	54.9	230	4.58	12.2	<100	2.10	199	1.0	2.29	16.2	0.624
Northwest Africa 11637	19	6	408	143	4.63	61.6	186	52.8	162	4.76	12.7	<60	2.10	171	1.1	2.33	15.9	0.638
Northwest Africa 3202	05	8	469	168	4.49	57.6	139	60.4	166	4.23	25.0	<170	2.85	876		11.5	28.8	0.580
Northwest Africa 3202	05	10	558	107	4.52	54.6	122	57.3	158	4.05	23.5	<150	2.79	830		11.3	27.9	0.560
Nova 057			485	36	4.65	67.0	120	50.7	211	7.55	13.6	<100	1.98	961	7.5	9.87	18.6	0.878
Pirapora	76	11	664	109	4.41	56.5	136	57.2		3.51			3.38	2880		27.2		0.535
Pirapora	77	1	432	111	4.55	56.3		60.0		3.74			3.51	2734		30.9		0.524
Pirapora	02	12	419	88	4.51	52.5	138	59.8	188	3.92		<40	3.55	3250		31.6	34.4	0.520
Santo Antonio	14	1		839	4.56	57.1	144	59.3	143	4.48	17.5	<150	2.67	415	2.2	6.01	23.7	0.585
Santo Antonio	14	8	545	201	4.55	58.1	131	60.9	137	4.74	18.5	<100	2.77	496	2.4	6.05	24.2	0.602
Uasara	10	5	565	73	4.43	54.3	145	59.9	154	3.76	32.6	<100	3.77	5820	68.5	48.0	35.8	0.473
Uasara	10	8	576	71	4.47	56.6	137	59.1	202	3.75	26.7	<150	3.69	5403	58.1	48.3	39.1	0.485
Xifu	14	5	553	19	4.70	58.4	169	54.1	122	8.88	3.6	<150	0.69	<50	0.2	0.023	4.1	1.035
Xifu	14	10	567	17	4.72	64.3	133	54.7	157	8.88	4.6	<150	0.81	<25	<0.2	0.024	4.2	1.051
Yamato 75105	78	2	217	45	4.85	60.8	141	55.9		4.72			1.90	301		2.84		0.700
**IIAB-an**																		
Elephant Moraine 83245	85	10	386	21	4.76	63.3	119	56.2		9.74			0.71	<140		0.026		1.078
**IVA**																		
Christiana	18	6	456	267	3.90	78.1	156	1.77		3.63	5.8		0.54	286	2.6	2.52	6.1	0.888
Huangling	81	7	597	395	3.82	76.0	137	1.88		2.29		37	0.84	401		3.41		0.629
Huangling	81	7		380	3.68	78.1	150	1.86	0.180	2.26		40	0.85	370		2.83		0.653
LAR 06877	08	3	769	19	4.08	86.8	117	2.27		10.4	3.0		0.38	79		0.83	4.1	1.981
LAR 06877	08	6	655	8.6	4.05	89.7	127	2.21		10.6	2.6		0.42	92		0.82	4.3	2.006
MIL 03356	05	10	577	20	4.07	88.2	101	2.17		9.59	4.1		0.39	92		1.00	4.3	1.983
MIL 07119	09	6	441	36	4.05	89.7	112	2.31		9.87	3.4		0.39	104		1.00	4.9	1.984
MIL 07119	10	1	590	101	4.06	90.1	107	2.19		10.3	3.3		0.46	101	0.88	0.99	4.5	2.009
Nova 019	18	6	444	260	3.89	75.3	142	2.16		3.64	3.8		0.59	302	2.7	2.49	5.0	0.917
Novorybinskoe	06	3	607	348	4.14	90.8	111	2.39		13.8	2.8		0.42	87		1.00	4.9	2.345
Novorybinskoe	06	5	555	195	4.15	95.3	105	2.31		13.1	3.2		0.39	116		1.01	3.9	2.401
Parkajoki 1	18	10	668	22	4.15	95.4	799	2.35		11.3	2.9		6.89	155	1.3	1.25	5.2	2.186
Parkajoki 2	18	6	539	41	4.11	95.0	603	2.12		11.4	4.4		1.30	200	2.60	1.26	5.2	2.199
Parkajoki 3	18	10	509	146	4.13	95.6	672	2.30		11.4	3.1		1.77	159	1.4	1.25	4.2	2.187
Parkajoki 4	18	6	602	24	4.09	97.8	640	2.52		11.8	2.9		1.68	128	1.47	1.28	5.2	2.213
Parkajoki 5	18	10	525	15	4.14	97.4	643	2.56		11.0	3.0		2.47	105	1.3	1.23	4.5	2.209
Parkajoki 6	18	6	490	17	4.19	103.4	668	2.62		11.3	3.3		2.57	103	1.27	0.96	3.3	2.271
Railways	15	9	611	697	3.86	78.9					4.4				2.9	2.49	6.9	0.877
Railways	16	4	628	158	3.91	80.4	162	1.98		3.60	4.0		0.51	287	2.5	2.54	5.5	0.882
Serrania de Varas	08	3	564	118	3.92	83.1	106	1.44		4.33	4.0		0.49	204		2.20	6.5	1.116
Serrania de Varas	07	11	651	130	3.89	80.1	143	2.21		5.05	3.9		0.70	261		2.17	5.5	1.196
**IVA-an**																		
Longchang	81	7	574	655	4.37	85.7	161	1.91		5.25		4824	1.26	568		5.21		0.538
Longchang	81	7		650	4.23	89.8	173	1.95		5.35		5336	1.32	560		4.50		0.573
Longchang	81	9	465	114	4.17	82.2	147	2.11		4.10		4702	1.16	465		4.09		0.616
Longchang	81	9		116	4.09	80.4	151	2.18		4.15		3695	1.33	500		3.73		0.671
Zaragoza	10	1	559	199	4.14	77.2	153	2.12		3.85	4.5		0.59	298	3.03	2.50	5.5	0.973
**IIIE**																		
Piedade do Bagre	82	5	903	220	4.65	77.5	146	15.0		2.49			1.58	1221		11.1		0.407
Piedade do Bagre	82	9	398	252	4.70	76.8	143	15.9		2.61			1.73	1241		11.5		0.412
Piedade do Bagre	13	7	368	315	4.66	72.9	152	15.7		2.53	12.1		1.69	1190	12.6	11.3	15.3	0.490
Cachiyuyal	82	9	569	194	4.78	80.3	148	16.9		4.56			1.42	253		2.62		0.553
Cachiyuyal	82	10	835	191	4.78	78.4	134	16.1		3.55			1.46	259		2.63		0.541
Cachiyuyal	05	4	401	202	4.75	80.1	132	16.0		3.47	10.9		1.32	240		2.70	11.6	0.543
Murfreesboro	82	12	421	202	4.74	77.7	142	16.9		3.09			1.55	151		2.14		0.504
Murfreesboro	83	3	386	113	4.72	77.0	145	16.6		3.26			1.52	154		1.85		0.492
Murfreesboro	14	10	506	191	4.74	78.6	148	17.7		3.42	9.5		1.43	163	1.01	2.22	13.6	0.502
Coopertown	96	5	388	140	4.82	83.9	138	16.3		4.74			1.70			0.597	6.4	0.691
Coopertown	96	8	516	148	4.85	83.8	150	16.3		4.31		167	1.66			0.603	10.1	0.696
Coopertown	16	1	563	131	4.88	79.5	130	17.1		4.81	7.7		1.08	82	0.3	0.590	7.5	0.687
Kokstad	04	4	620	73	4.90	79.5	136	16.5		4.17	6.5		1.02	45		0.579	8	0.676
Kokstad	04	11	584	88	4.81	82.9	140	17.8		4.60	10.0		1.10	52		0.577	8.6	0.675
Aliskerovo	85	6	755	82	4.86	83.3	145	17.3		4.66		821	1.08			0.488		0.701
Aliskerovo	85	8	690	79	4.75	79.6	139	17.0		4.15		76	1.05	38		0.488		0.725
Aliskerovo	96	5	323	92	4.93	83.9	140	18.1		4.89			1.19			0.551	9.3	0.775
Aliskerovo	96	10	568	75	4.89	84.4	133	18.0		4.47			1.01			0.492	7.9	0.698
Burlington	82	12	382	605	4.85	81.3	131	18.3		5.40			1.15			0.508		0.759
Burlington	83	3	468	319	4.83	82.7	139	17.7		5.28			1.03	44		0.439		0.720
Burlington	13	7	352	590	4.88	86.8	148	18.8		5.39	6.4	338	1.07	51		0.540	8.7	0.764
Paneth's Iron	82	12	878	104	4.95	82.7	142	18.5		5.85			1.16			0.399		0.831
Paneth's Iron	96	5	637	47	4.94	81.3	132	17.3		5.36		249	0.93			0.399	8.5	0.696

Paneth's Iron	99	5	495	46	4.88	81.5	132	17.7		5.54			0.90			0.408	7.0	0.789
Tanokami Mountain	96	12	463	39	4.98	84.4	163	18.3		6.24			0.78			0.182	7.3	0.887
Tanokami Mountain	98	8	565	54	5.03	84.4	138	16.8		5.98			0.74	73		0.167	7.7	0.886
Northwest Africa 4704	07	4	449	37	4.88	88.0	153	16.5		4.56	5.0		0.76			0.148	5.5	0.898
Northwest Africa 4704	07	6	451	40	4.89	89.9	155	18.5		5.41	4.9		0.82			0.150	5.9	0.828
Porto Alegre	06	7	647	74	4.96	93.2	146	19.2		6.02	7.2		0.84			0.153	6.7	0.939
Porto Alegre	06	12	467	67	4.96	87.1	146	17.1	58	5.28	8.8		0.76			0.143	5.4	0.857
Staunton	96	5	505	67	4.93	84.6	102	16.8		5.20			0.83	50		0.127	7.5	0.881
Staunton	96	10	537	74	5.00	86.5	123	18.2		5.66			0.74			0.127	5.8	0.850
Paloduro	80	8	670	136	4.88	86.0	127	18.1		5.61	nd		0.79			0.118	nd	0.852
Paloduro	81	11	796	187	4.89	88.5	142	17.8		5.71	nd		0.80			0.127	nd	0.847
Paloduro	97	5	635	178	4.91	84.5	117	17.3		5.57			0.71			0.121	7.9	0.852
Paloduro	13	2	511	296	4.87	81.5	129	18.4		6.06	3.6		0.80			0.109	4.8	0.825
Rhine Villa	96	10	675	50	4.86	89.9	133	19.1		5.42			0.75	90		0.116	6.6	0.871
Rhine Villa	96	12	784	58	4.84	87.1	134	17.8		5.39			0.73			0.114	6.7	0.878
Willow Creek	96	10	445	40	5.02	86.8	125	19.9		6.88			0.76			0.068	5.3	0.970
Willow Creek	96	12	462	50	4.82	91.5	129	18.9		6.62			0.59			0.067	4.8	0.970
Colonia Obrera	96	12	270	42	4.85	94.1	153	19.4		7.62			0.84			0.061	6.4	1.084
Colonia Obrera	97	5	552	28	4.95	85.5	128	18.5		7.45			0.59			0.057	4.2	1.041
**IIIE-an**																		
Aletai [Ulasitai]	16	1	586	45	5.27	89.7	113	16.8		14.7	1.5		0.32		0.11	0.242	1.6	1.967
Aletai [Ulasitai]	16	4	624	30	5.18	100.5	106	16.7		15.1	2.6		0.26	41		0.231	2.0	1.858
Aletai [Wuxilike]	14	5	665	53	5.21	97.4	102	17.2		16.0	2.5	256	0.25	12	0.50	0.237	3.5	1.868
Aletai [Wuxilike]	14	10	600	37	5.18	98.6	108	16.7		15.5	2.6		0.28	20	0.21	0.231	1.6	1.857
Aletai [Xinjiang (b)]	80	1	505	883	5.17	91.3	115	18.2		15.2			0.30			0.246		1.828
Aletai [Xinjiang (b)]	81	7	505	22	5.14	97.2	106	18.1		15.1			0.44			0.237		1.858
Aletai [Akebulake]	15	9	614	51	5.16	98.6	112	17.0		15.5		87	0.36			0.226	1.9	1.841
Aletai [Akebulake]	16	1	527	49	5.18	99.1	109	16.8		14.7	1.7		0.28		0.20	0.223	2.2	1.786

Table S4. Model-derived bulk compositions of groups IC, IIAB, IIIAB, IVA and IIIE. The optimal composition for each group is denoted by bold text. Concentrations in µg/g unless noted.

Group	P (wt.%)	S (wt.%)	Fe (mg/g)	Co (mg/g)	Ni (mg/g)	Cu	Ga	Ge	As	Mo	Ru	Rh	Pd	Sb† (ng/g)	W	Re (ng/g)	Os	Ir	Pt	Au
	0.48	13	806.7	3.10	55.0	280	28.0	110.0	6.20	5.4	5.5	0.90	2.5	330	0.80	250	3.0	2.80	8.0	0.68
	0.48	14	797.8	2.97	54.0	300	27.0	105.0	6.30	5.7	4.5	0.83	2.6	350	0.65	200	2.0	2.00	5.5	0.68
IC	**0.49**	**15**	**787.8**	**2.85**	**54.0**	**320**	**25.5**	**98.0**	**6.30**	**5.5**	**4.2**	**0.80**	**2.6**	**370**	**0.65**	**150**	**1.8**	**1.70**	**5.0**	**0.65**
	0.43	16	779.5	2.72	53.0	320	24.0	98.0	5.90	5.0	4.0	0.72	2.5	380	0.50	150	1.8	1.80	5.0	0.62
	0.45	17	769.4	2.60	53.0	370	20.5	89.0	5.90	5.0	3.5	0.65	2.5	370	0.50	100	0.8	1.10	3.6	0.63
	0.60	14	802.1	2.95	48.5	275	26.0	82.0	6.00		7.3		2.1	198	0.90	400	5.5	5.00	8.0	0.61
IIAB	**0.60**	**15**	**793.2**	**2.85**	**47.5**	**285**	**24.0**	**75.0**	**6.00**		**5.8**		**2.1**	**210**	**0.85**	**300**	**4.0**	**4.00**	**7.0**	**0.60**
	0.50	16	785.1	2.72	46.8	300	23.0	72.0	5.50		5.8		2.1	210	0.80	400	4.0	4.50	6.7	0.58
	0.35	*8*	*839*	*4.10*	*73.6*	*239*	*14.6*	*29.3*	*8.4*		5.5		3.2	*173*	*0.73*	*379*	*3.6*	*3.0*	*7.4*	*0.90*
IIIAB*	***0.30***	***9***	***830***	***3.90***	***72.7***	***248***	***13.6***	***27.1***	***7.4***		**6.0**		**3.0**	***143***	***0.66***	***358***	***3.4***	***3.7***	***6.7***	***0.87***
	0.30	*10*	*819*	*3.80*	*73.8*	*268*	*12.8*	*25.6*	*7.9*		5.0		3.2	*168*	*0.59*	*351*	*3.5*	*2.8*	*6.0*	*0.86*
	0.48	5	866.3	4.10	74.5	185	12.7	23.0	4.20	5.6	8.0	1.02	2.8	102	0.90	350	4.5	4.00	9.0	0.60
	0.45	6	856.7	4.00	74.5	185	12.7	23.0	4.40	5.3	8.0	0.98	2.9	109	0.90	350	4.5	4.00	9.0	0.60
IIIE	**0.50**	**7**	**845.8**	**3.92**	**75.0**	**195**	**12.6**	**23.5**	**4.80**	**6.0**	**7.0**	**0.95**	**3.0**	**120**	**0.75**	**350**	**4.0**	**3.50**	**8.0**	**0.65**
	0.50	8	835.9	3.80	75.0	210	12.0	22.5	5.00	6.0	6.3	0.90	3.1	132	0.75	310	3.5	3.30	8.0	0.65
	0.50	9	826.0	3.75	75.0	220	12.0	22.5	5.20	6.0	5.7	0.85	3.2	143	0.75	280	2.8	3.00	7.0	0.68
	0.13	2	901.9	3.63	73.0	150	1.75	0.094	4.40	5.2	4.5	0.74	3.7	4.3	0.52	380	4.0	3.20	5.3	0.92
IVA	**0.12**	**3**	**892.1**	**3.50**	**73.0**	**150**	**1.76**	**0.094**	**4.50**	**5.2**	**4.5**	**0.74**	**3.7**	**4.5**	**0.57**	**350**	**4.0**	**2.75**	**6.0**	**0.98**
	0.11	4	882.8	3.45	72.5	162	1.70	0.092	4.60	5.1	4.3	0.73	3.8	4.5	0.50	350	4.0	2.90	5.5	0.95

*Except for Ru and Pd, other data (italics) are from Ref (7).

†Bulk Sb data are from fewer INAA measurements.

Table S5. Bulk compositions of CC-iron groups and grouplet used in Figures 2 and S7. Concentrations in μg/g unless noted.

Group/ Grouplet	P (wt.%)	S (wt.%)	Fe (mg/g)	Co (mg/g)	Ni (mg/g)	Cu	Ga	Ge	As	Mo	Ru	Rh	Pd	Sb† (ng/g)	W	Re (ng/g)	Os	Ir	Pt	Au
IIC*	2.2	6	812.9	4.75	100.0	320	25.4	71.0	15.2	13.0	5.9	1.40	5.4	860	1.50	310	3.3	3.20	6.8	1.35
IID*	1.9	~0	865.6	6.80	108.0	235	75.9	97.0	11.6		15.0		4.4	290	3.00	1200	15.0	12.4	16.0	1.10
IIF*	0.70	5	817.2	6.15	119.0	430	7.6	110.0	12.5	13.0	12.5	1.80	5.2	170	0.90	830	9.5	9.00	15.0	1.10
IIIF†	1.2	2.0	890.8	3.07	73.0	115	6.674	0.965	4.28	8.0	8.0	1.20	4.0	75	1.20	685	8.0	6.00	8.0	0.70
IVB*	0.47	~0	809.7	7.87	177.5	5	0.237	0.07	1.45	34.0	26.0	3.70	9.45	8.2	3.10	2100	28.5	20.0	31.0	0.16
SBT*	1.5	8	716.7	7.60	180.0	550	11.8	32.0	17.2	20.0	11.5	2.96	8.4	580	0.40	670	7.3	7.30	12.7	1.72

*Data from Ref (11)

†Data from Ref (22)

SI References

1. N. L. Chabot, J. H. Jones, The parameterization of solid metal-liquid metal partitioning of siderophile elements. *Meteoritics and Planetary Science* **38**, 1425-1436 (2003).
2. J. Willis, J. I. Goldstein, The effects of C, P, and S on trace element partitioning during solidification in Fe-Ni alloys. *Journal of Geophysical Research: Solid Earth* **87**, A435-A445 (1982).
3. D. J. Malvin, J. H. Jones, M. J. Drake, Experimental investigations of trace element fractionation in iron meteorites. III: Elemental partitioning in the system Fe-Ni-SP. *Geochim. Cosmochim. Acta* **50**, 1221-1231 (1986).
4. J. H. Jones, M. J. Drake, Experimental investigations of trace element fractionation in iron meteorites, II: The influence of sulfur. *Geochim. Cosmochim. Acta* **47**, 1199-1209 (1983).
5. N. L. Chabot, Sulfur contents of the parental metallic cores of magmatic iron meteorites. *Geochim. Cosmochim. Acta* **68**, 3607-3618 (2004).
6. J. T. Wasson, Trapped melt in IIIAB irons; solid/liquid elemental partitioning during the fractionation of the IIIAB magma. *Geochim. Cosmochim. Acta* **63**, 2875-2889 (1999).
7. N. L. Chabot, B. Zhang, A revised trapped melt model for iron meteorites applied to the IIIAB group. *Meteorit. Planet. Sci.* **57**, 200-227 (2021).
8. N. L. Chabot, E. A. Wollack, W. F. McDonough, R. D. Ash, S. A. Saslow, Experimental determination of partitioning in the Fe-Ni system for applications to modeling meteoritic metals. *Meteorit. Planet. Sci.* **52**, 1133-1145 (2017).
9. J. H. Jones, D. J. Malvin, A nonmetal interaction model for the segregation of trace metals during solidification of Fe-Ni-S, Fe-Ni-P, and Fe-Ni-SP alloys. *Metallurgical Transactions B* **21**, 697-706 (1990).
10. V. F. Buchwald, *Handbook of iron meteorites. Their history, distribution, composition and structure* (Arizona State University, 1975).
11. B. Zhang, N. L. Chabot, A. E. Rubin, Compositions of carbonaceous-type asteroidal cores in the early solar system. *Science Advances* **8**, eabo5781 (2022).
12. A. J. Campbell, M. Humayun, Compositions of group IVB iron meteorites and their parent melt. *Geochim. Cosmochim. Acta* **69**, 4733-4744 (2005).
13. J. Wasson, J. Richardson, Fractionation trends among IVA iron meteorites: Contrasts with IIIAB trends. *Geochim. Cosmochim. Acta* **65**, 951-970 (2001).
14. J. T. Wasson, G. W. Kallemeyn, Compositions of chondrites. *Philosophical Transactions of the Royal Society of London. Series A, Mathematical and Physical Sciences* **325**, 535-544 (1988).
15. G. W. Kallemeyn, A. E. Rubin, J. T. Wasson, The compositional classification of chondrites: V. The Karoonda (CK) group of carbonaceous chondrites. *Geochim. Cosmochim. Acta* **55**, 881-892 (1991).
16. G. W. Kallemeyn, A. E. Rubin, J. T. Wasson, The compositional classification of chondrites: VI. The CR carbonaceous chondrite group. *Geochim. Cosmochim. Acta* **58**, 2873-2888 (1994).
17. A. E. Rubin, Carbonaceous and noncarbonaceous iron meteorites: Differences in chemical, physical, and collective properties. *Meteorit. Planet. Sci.* **53**, 2357-2371 (2018).
18. D. Wark, W. V. Boynton, The formation of rims on calcium-aluminum-rich inclusions: Step I—Flash heating. *Meteorit. Planet. Sci.* **36**, 1135-1166 (2001).
19. G. Consolmagno, D. Britt, R. Macke, The significance of meteorite density and porosity. *Geochemistry* **68**, 1-29 (2008).
20. C. M. Wai, J. T. Wasson, Nebular condensation of moderately volatile elements and their abundances in ordinary chondrites. *Earth Planet. Sci. Lett.* **36**, 1-13 (1977).
21. K. Lodders, Solar system abundances and condensation temperatures of the elements. *The Astrophysical Journal* **591**, 1220 (2003).
22. B. Zhang *et al.*, Chemical study of group IIIF iron meteorites and the potentially related pallasites Zinder and Northwest Africa 1911. *Geochim. Cosmochim. Acta* **323**, 202-219 (2022).
23. A. E. Rubin, B. Zhang, N. L. Chabot, IVA iron meteorites as late-stage crystallization products affected by multiple collisional events. *Geochim. Cosmochim. Acta* **331**, 1-17 (2022).
24. B. J. Wood, D. J. Smythe, T. Harrison, The condensation temperatures of the elements: A reappraisal. *Am. Mineral.* **104**, 844-856 (2019).
25. H. A. Tornabene, R. D. Ash, R. J. Walker, K. R. Bermingham, Genetics, age, and crystallization history of group IC iron meteorites. *Geochim. Cosmochim. Acta* **340**, 108-119 (2023).
26. C. D. Hilton, R. D. Ash, R. J. Walker, Chemical characteristics of iron meteorite parent bodies. *Geochim. Cosmochim. Acta* **318**, 112-125 (2022).

27. E. Chiappe (2022) Genetics, Ages, and Chemical Compositions of the Group IIIE Iron Meteorites and the Iron Meteorite Lieksa. (University of Maryland, College Park).
28. J. T. Wasson, H. Huber, D. J. Malvin, Formation of IIAB iron meteorites. *Geochim. Cosmochim. Acta* **71**, 760-781 (2007).
